# Development of Hydrogen Sulfide-Releasing Carbonic Anhydrases IX- and XII-Selective Inhibitors with Enhanced Antihyperalgesic Action in a Rat Model of Arthritis

**DOI:** 10.1021/acs.jmedchem.2c00982

**Published:** 2022-09-19

**Authors:** Alessandro Bonardi, Laura Micheli, Lorenzo Di Cesare Mannelli, Carla Ghelardini, Paola Gratteri, Alessio Nocentini, Claudiu T. Supuran

**Affiliations:** † Department of NEUROFARBA—Pharmaceutical and Nutraceutical Section, University of Firenze, via Ugo Schiff 6, Sesto Fiorentino, 50019 Florence, Italy; ‡ Department NEUROFARBA—Section of Pharmacology and Toxicology, University of Florence, viale Gaetano Pieraccini 6, Firenze, 50139 Florence, Italy

## Abstract

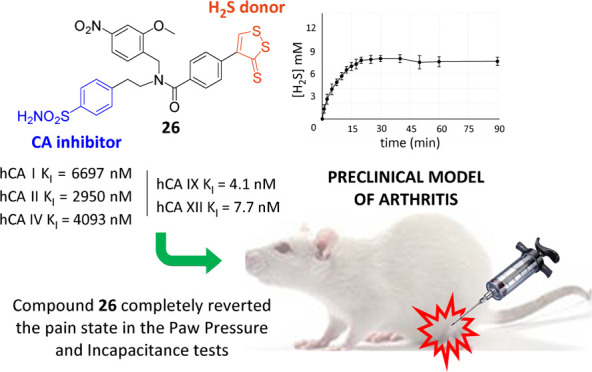

An effective therapeutic approach based on the anti-inflammatory action of hydrogen sulfide (H_2_S) and inhibition of carbonic anhydrases (CAs) IX and XII is proposed here for the management of arthritis. H_2_S is a human gasotransmitter that modulates inflammatory response at low concentrations. Inhibition of CAs IX and XII can repristinate normal pH in the acidic inflamed synovial fluid, alleviating arthritis symptoms. We report here the design of H_2_S donor—CA inhibitor (CAI) hybrid derivatives. The latter were tested in vitro as inhibitors of human CAs I, II, IV, IX, and XII, showing a markedly increased inhibition potency/isoform selectivity compared to the CAI synthetic precursors. The best compounds demonstrated the ability to consistently release H_2_S and produce a potent pain-relieving effect in a rat model of arthritis. Compound **26** completely reverted the pain state 45 min after administration with enhanced antihyperalgesic effect in vivo compared to the single H_2_S donor, CAI fragment, or their co-administration.

## Introduction

Rheumatoid arthritis (RA) and juvenile idiopathic arthritis (JIA) are inflammatory diseases affecting people over sixty and under sixteen years old, respectively, characterized by chronic inflammation of the synovial membrane, that leads to articular cartilage and juxta-articular bone destruction and, eventually, deformity. Both conditions are accompanied by pain.^1−3^ The pathophysiology of RA and JIA is unknown to date but was shown to be related to the previous activation of endothelial cells with the new blood vessel growth and a hyperplastic expansion of the synovial membrane that invades the periarticular bone at the cartilage–bone junction, leading to bone erosions and cartilage degradation.^1−3^ This process promotes the migration *in loco* of macrophages and leukocytes (T cells, B cells, and monocytes) that release pro-inflammatory cytokines, such as the tumor necrosis factor and interleukin-6 (IL-6), stimulating the production of several secondary modulators: the nuclear factor κB ligand (RANKL), which is responsible for the osteoclast differentiation and osteoblasts apoptosis, prostaglandins, and matrix metalloproteinases, that mediate the symptoms of the disease including pain, swelling, and degradation of cartilage and bone.^1,2,4^

An important piece of evidence is that the patients with RA showed the concomitant presence of a lower pH (6.5) in the synovial fluid compared to healthy patients or affected by osteoporosis, which is linked to inflammatory reaction and direct damage to the cartilage and tissue around the joint.^5−8^ Furthermore, it was demonstrated that the synovial fluid becomes acidic as the intensity of the inflammatory response increases, and the more intense the inflammatory reaction was the more acidic the synovial fluid pH became, provoking pain.^5^

Several isoforms of the metalloenzymes carbonic anhydrases (Cas and EC 4.2.1.1), that catalyzed the reversible hydration of carbon dioxide into bicarbonate and proton ions, have been associated with articular inflammatory diseases.^9,10^ In detail, CA I was found to be overexpressed in the synovium of the patients with ankylosing spondylitis, and transgenic mice that overexpressed CA I showed aggravated joint inflammation and destruction.^11,12^ Furthermore, antibodies to CA III and IV have been identified in RA.^8^ The latter study also showed that CA activity in RA was significantly higher than that of control groups. Most importantly, in 2016, Cimaz et al. demonstrated that isoforms CA IX and XII, which are usually related to hypoxic tumors, are also overexpressed in the inflamed synovium of patients affected by JIA.^13^

Hydrogen sulfide is the third gasotransmitter of the human body, along with nitric oxide (NO) and carbon monoxide (CO), which have been widely investigated for biomedical purposes over the last three decades.^14^ Its endogenous synthesis is attributed to three enzymes with different subcellular and tissue distribution called cystathionine-β-synthase, cystathionine-γ-lyase, and 3-mercaptopyruvate sulfurtransferase, which use l-cysteine, l-homocysteine, and 3-mercaptopyruvate, respectively, as the substrate.^14−17^ Once synthesized by a cell, this ubiquitous small gaseous signaling molecule performs a paracrine action that involves at least 200 neighboring cells, thanks to its lipophilic nature which allows it to cross the membranes.^18^ Hydrogen sulfide has not a specific receptor or signaling pathway, but it triggers many cellular effectors in a cell/tissue/species-dependent way, being involved in various and important physiological processes such as modulation of vascular tone and blood pressure, neurotransmission and nociception, angiogenesis, cardiac function, various leukocytic functions, penile erectile function, scavenger action, acting as an adenosine 5′-triphosphate (ATP) synthesis stimulator, and so forth.^14,18^

Numerous studies demonstrated that the pathogenesis of many diseases, including several inflammatory disorders, is related to hydrogen sulfide deficiency, and the treatment with H_2_S-donating molecules improves symptoms.^14,19−28^

Based on this evidence, hydrogen sulfide donor—CA inhibitor hybrid derivatives are here proposed to combine the action of H_2_S and inhibitors of specific human (h) CA isoforms for the treatment of inflammation, specifically RA. In fact, inhibition of the hCA isoforms, whose activity is abnormal on the synovial membrane, was shown to repristinate the normal pH of the synovial fluid, alleviating the RA and JIA symptoms (pain, cartilages, and bony erosion).^29−33^ On the other hand, the released H_2_S suppresses the inflammatory process on multiple levels; improves symptoms such as redness, swelling, heat, pain, and loss of function, blocking leucocyte adhesion, and promoting neutrophils apoptosis, analgesia, and reparation of the damage.^14,19^ Moreover, H_2_S prevents the formation of the complex RANKL-RANK (ligand–receptor activator of nuclear factor kappa-Β), blocking the transcription of pro-inflammatory cytokines, osteoclast differentiation, and osteoblast apoptosis, protecting bone erosion.^4,22,34^ A similar study was recently reported by some of us over the development of effective CAIs incorporating scaffolds that release carbon monoxide (based on the dicobalt hexacarbonyl complex), another gasotransmitter showing pharmacological application for the management of inflammation.^30^

## Results and Discussion

### Drug Design and Chemistry

**Figure 1:**
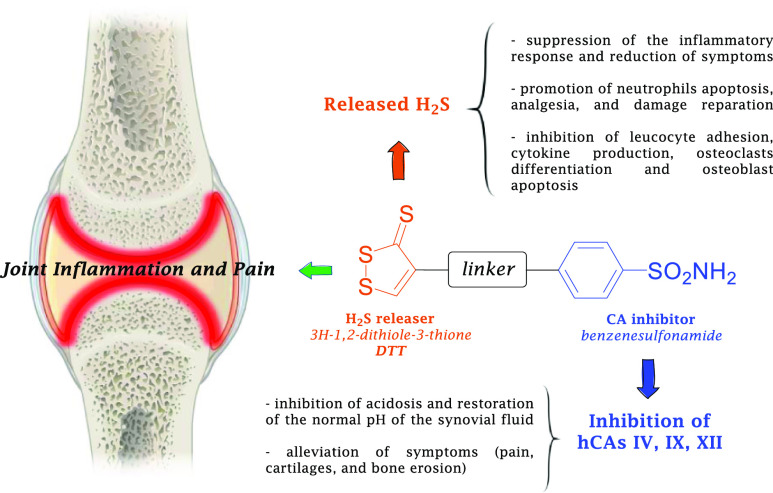
Rational design of H_2_S releaser—CAI hybrid derivatives for the management of inflammation.

The design of H_2_S donor—CAI hybrids took into account that H_2_S is also a rather toxic gas. In fact, if inhaled at concentrations of >20 ppm, it enters the bloodstream and tissues determining the blockage of the cell respiratory chain by binding the cytochrome C oxidase iron, inducing oxidative stress, and blocking the synthesis of ATP, which is fatal at a concentration of 1000 ppm.^15^ For these reasons, it is important to control the H_2_S release in order to achieve an optimal concentration for the therapeutic effect and not poisoning. Among the numerous H_2_S-releasing scaffolds reported in the literature, which showed different gasotransmitter releasing properties, the drug design strategy here proposed was initiated with the 4-substituted 3*H*-1,2-dithiole-3-thione chemotype (**DTT**, Figure 1),^35^ chosen on the basis of the target therapeutic action and resulting necessary H_2_S release rate. As for including CAI scaffolds into the molecular hybrids, the choice fell on the benzenesulfonamide chemotype which furnishes optimal hCA inhibition potency and was shown to be suitable for drug design strategies aimed at enhancing isoform selectivity.^36,37^

**Scheme 1:**
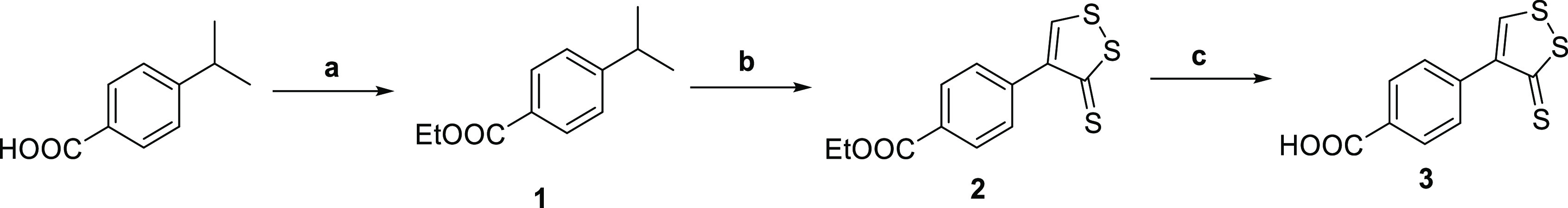
Synthesis of the Intermediate 3*H*-1,2-Dithiole-3-thione **3** Reagents and conditions: (a) SOCl_2_, dry EtOH, 0 → 60 °C, 6 h, 92%; (b) S_8_, 135 → 220 °C, 6–8 h, 75%; (c) H_2_SO_4_ 9 M, CH_3_COOH, 100 °C, 4 h, 81%.

The synthesis of the H_2_S releaser—CAI hybrids starts with the preparation of the 3*H*-1,2-dithiole-3-thione core, which bears a *p*-COOH-phenyl moiety at position 4 for the subsequent hybridization with the CAI scaffold. Initially, 4-isopropylbenzoic acid was protected as ethyl ester **1**, using SOCl_2_ in dry ethanol (EtOH) (Scheme 1). The cyclization to 3*H*-1,2-dithiole-3-thione was obtained by treating derivative **1** in melted sulfur at 220 °C, to give intermediate **2**, whose ester group was thus hydrolyzed with H_2_SO_4_ in CH_3_COOH at 100 °C, yielding the carboxylic acid **3**.

**Scheme 2:**
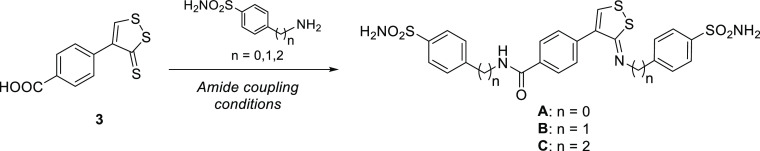
Formation of Side Products **A–C** Upon Amide Coupling of Carboxylic Acid **3** with Primary Amine Benzenesulfonamide Derivatives

The coupling reaction of **3** with primary amine derivatives of benzenesulfonamides [e.g. sulfanilamide, 4-(aminomethyl)benzenesulfonamide, 4-(2-aminoethyl)benzenesulfonamide] with several coupling agents and reaction conditions unexpectedly always led to the undesired attack on the scaffold thione group with the formation of the imine-like derivatives **A–C** even at very low temperatures (Scheme 2).

**Scheme 3:**
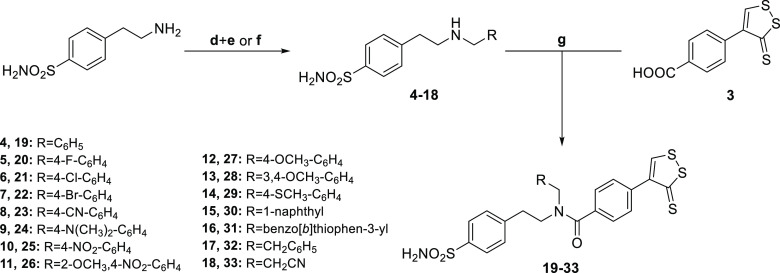
Synthesis of 3*H*-1,2-Dithiole-3-thione Benzenesulfonamide Hybrid Derivatives **19–33** Reagents and conditions: (d) RCHO, dry MeOH, reflux, 4 h; (e) NaBH_4_, dry MeOH, reflux, 0.5–2 h; (f) R–X, dry DMF, r.t or 60 °C, 0.5–6 h; (g) DIPEA, PyBOP, dry DMF; r.t, overnight (o.n.).

As a result, it was supposed that the use of secondary amines benzenesulfonamide derivatives, such as **4–18**, could prevent the formation of these undesired side products in the coupling reaction conditions used in our experiments (Scheme 3). Hence, 4-(2-aminoethyl)benzenesulfonamide was treated by reductive amination in presence of the proper aromatic aldehydes and sodium borohydride in dry methanol (MeOH) or, alternatively, by nucleophilic substitution with the appropriate halides in dry dimethylformamide (DMF), to give secondary amines **4–18**. The latter was coupled with carboxylic acid **3** by using PyBOP as the coupling agent and DIPEA as a base in dry DMF at room temperature (r.t.).

### CA Inhibition

The synthesized compounds **19–33** were tested for their inhibitory action against hCA isoforms I, II, IV, IX, and XII, by a stopped-flow kinetic assay using acetazolamide (**AAZ**) as standard.^38^ The cytosolic hCA I and II are considered off-target for the CAI anti-inflammatory therapeutic application because they are ubiquitous and responsible for most CAI side effects. In contrast, hCAs IV, IX, and XII were the target isoforms being overexpressed on the synovial membrane and linked to inflamed conditions.^29−33,39^

Table 1 gathers the inhibition constants (*K*_I_s) of compounds **19–33** against the panel of hCAs, whereas the selectivity index (SI) against hCA IV, IX, and XII over the off-target isoforms hCA I and II are collected in Table 2.

**Table 1:**
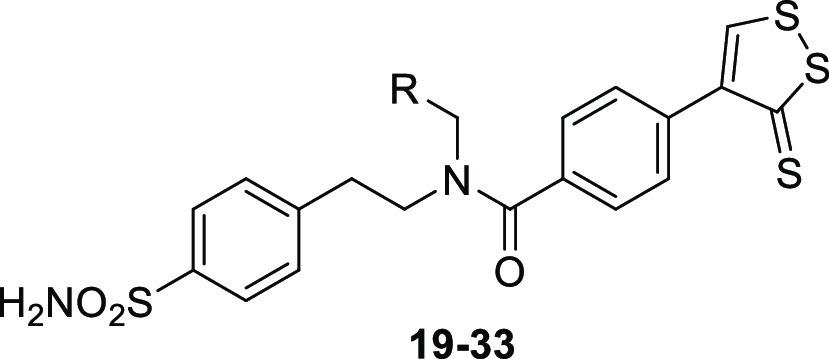

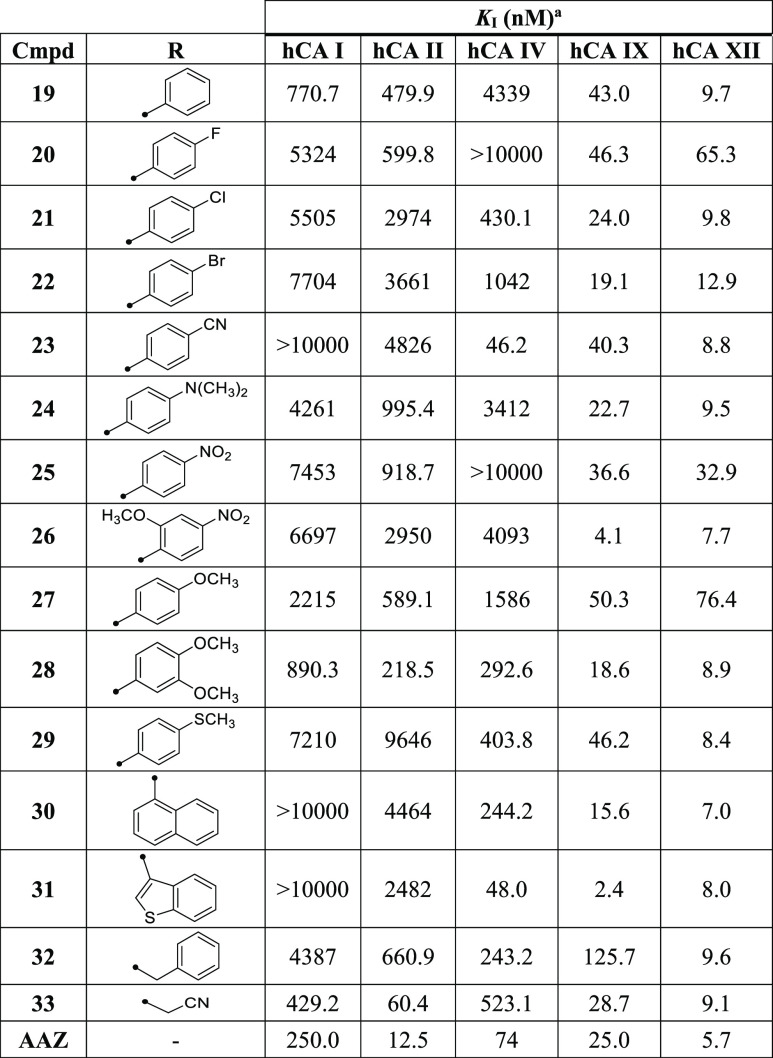
Inhibition Data of Human CA Isoforms hCA I, II, IV, IX, and XII with Sulfonamides **19–33** Reported Here and the Standard Sulfonamide Inhibitor **AAZ** by a Stopped-Flow CO_2_ Hydrase Assay^38^

a Mean from three different assays, by a stopped-flow technique (errors were in the range of ±5–10% of the reported values).

**Table 2:** SI of Sulfonamides **19–33** and Standard **AAZ** Against the anti-inflammatory Human CA Isoforms IV, IX, and XII versus the Ubiquitous Cytosolic Ones CA I and II

	SI					
cmpd	CA I/IV	CA II/IV	CA I/IX	CA II/IX	CA I/XII	CA II/XII
**19**	0.18	0.11	19.2	11.1	79.4	49.5
**20**	<0.53	<0.06	115	13.0	81.5	9.2
**21**	12.8	6.9	229	124	561	303
**22**	7.4	3.5	403	192	597	284
**23**	>216.4	104	>248	120	>1136	548
**24**	1.2	0.3	188	43.9	448	105
**25**	<0.7	<0.09	204	25.1	226	27.9
**26**	1.6	0.7	1633	719	870	383
**27**	1.4	0.4	44.0	11.7	29.0	7.7
**28**	3.0	0.7	47.9	11.7	105	25.7
**29**	17.9	23.9	156	209	858	1148
**30**	>41.0	18.3	>641	286	>1428	637
**31**	>208	51.7	>4167	103	>1204	299
**32**	18.0	2.7	34.9	5.3	462	69.6
**33**	0.8	0.11	15.0	2.1	45.2	6.4
**AAZ**	3.4	0.17	10.0	0.5	43.9	2.2

Most tested compounds displayed weak inhibition against the off-target hCA I, with *K*_I_ values in a rather flat micromolar range (*K*_I_s of 2215–10000 μM), except derivatives **19** and **28** that inhibited this ubiquitous isoform in the high nanomolar range (*K*_I_ of 770.7–890.3 nM). In contrast, all tested compounds produced a greater hCA II inhibition when compared to hCAI (*K*_I_s in the range 218.5–4826 nM), with the exception of compound **29**. In fact, derivatives **19**, **20**, **27**, **28**, and **32** inhibit hCA II significantly below the micromolar range (*K*_I_s of 218.5–660.9 nM). It should be noted that the elongation of the N-substituent from a benzyl (**19**) to a phenethyl (**32**) decreases significantly the inhibitory action against hCA I (*K*_I_ from 770.7 to 4387 nM) and with minor extent against hCA II (*K*_I_ from 479.9 to 660.9 nM). Interestingly, the only tertiary amide derivative not bearing an aromatic scaffold on the N-substituent, that is the nitrile **33** (*R* = CH_2_CN), shows the greatest efficacy against hCAs I and II (*K*_I_s of 429.2 and 60.4 nM, respectively). It can be speculated that the loss of steric encumbrance of **33** in comparison to other derivatives favors the ligand binding in the narrow active sites of these ubiquitous isoforms. As a matter of fact, all substituents included on the outer phenyl ring of compound **19** decrease the inhibitory action against the cytosolic isoforms, with the only exception being the dimethoxy derivative **28** against hCA II (*K*_I_ of 479.9 vs 218.5 nM).

As for hCA IV, the *p*-CN-phenyl and benzo[*b*]thiophen-3-yl derivatives **23** and **31** only exhibit *K*_I_ values below 100 nM (K_I_s of 46.2 and 48.0 nM), resulting in the most potent and also the first and the second most selective compounds against hCA IV over hCA II (SI of 104 and 52, respectively).

Compounds **21**, **28–30**, **32**, and **33** produce instead hCA IV inhibition in the high nanomolar range (*K*_I_s of 243.2–523.1 nM), while inhibitors **19**, **22**, **26**, and **27** acted in the low micromolar span (*K*_I_s of 1042–4339 nM)and up to no inhibition detected below 10 μM for **20** and **25**.

Notably, hCA IX and XII were the isoforms most inhibited by all 3*H*-1,2-dithiole-3-thione derivatives, with most *K*_I_s collected in a narrow range for both isoforms (*K*_I_s spanning between 2.4–50.3 nM and 5.7–32.9 nM for hCA IX and XII, respectively). It should be stressed that the target isozymes IX and XII show in fact rather roomier active sites when compared to other hCAs, which can favor the accommodation and binding of the bulky tertiary amide derivatives **19–32** after the binding of the sulfonamide moiety to the zinc ion. In particular, compounds **31** and **26** were the most potent and selective hCA IX inhibitors with a single digit (*K*_I_s of 2.4 and 4.1 nM, respectively, and hCA II/hCA IX SI of 1034 and 719, respectively). As with hCAs I and II, the elongation of the N-substituent from **19** to **32** led the *K*_I_ above 100 nM (*K*_I_ = 125.7 nM). All other hybrid compounds exhibited medium nanomolar range hCA IX inhibition with *K*_I_ values between 15.6 and 50.3 nM.

On the other hand, hCA XII was inhibited with single-digit *K*_I_s by a greater subset of compounds (**19**, **21**, **23**, **24**, **26**, and **28–33**) (*K*_I_s in the range 7.0–9.8 nM). Among these, derivatives **30** (*K*_I_ = 7.0 nM) and **26** (*K*_I_ = 7.7 nM) were the most potent hCA XII inhibitors, while **29** (*K*_I_ = 8.4 nM) and **30** (*K*_I_ = 7.0 nM) were the most selective hCA XII inhibitors over the main off-target hCA II (SI of 1148 and 637, respectively). Compounds **20**, **22**, **25,** and **27** inhibited instead hCA XII in the medium nanomolar range with *K*_I_ values between 12.9 and 76.4 nM.

Overall, the obligatory choice of coupling bulky secondary instead of primary amine benzenesulfonamide derivatives to the H_2_S donor nucleus, due to side products formation in the planned synthetic pathway, resulted to be a good option for the achievement of potent hCA IX and XII inhibitors also showing a high selectivity over off-target hCAs. In fact, the incorporation of N-alkyl aromatic substituents led the 3*H*-1,2-dithiole-3-thione derivatives to prefer the binding to the roomier active site of hCA IX and XII with respect to other hCA isoforms. In fact, it can be noted that the 2-ethylcyano derivative **33** showing the least steric encumbrance within the series exhibited a significant loss of target/off-target selectivity of action in comparison to most other such hybrid compounds, with the hCA II/IX SI being only 2.1.

It should be also stressed that the secondary amine benzenesulfonamide derivatives **4–18** do not show notable CA inhibition profiles both in terms of potency and isoform selectivity, as previously reported,^32,33^ and here further investigated. Table S1, Supporting Information collects the inhibition profiles of **4–18** against hCA I, II, IV, IX, and XII, while the related SI values were reported in Table S2, Supporting Information. Generally, benzenesulfonamide derivatives **4–18** were medium to high nanomolar inhibitors of hCA I (*K*_I_ = 82.3–373.8 nM), II (*K*_I_ = 47.3–201.6 nM), IX (45.3–119.9), and XII (*K*_I_ = 46.2–113.2 nM), and weak inhibitors of hCA IV with inhibition constant (*K*_I_) values in the low micromolar range (0.9–7.2 μM). The 1-naphthyl derivative **15** stood out as the most promising one, according to inhibition potency (hCA IX, *K*_I_s of 45.3; hCA XII, 54.7 nM) and selectivity against hCAs IX and XII over off-target isoforms (CA I/IX SI = 5.8; CA I/XII SI = 4.8; CA II/IX SI = 4.0; and CA II/XII SI = 3.2). It is worth noting that, as previously shown,^35,36^ most such single CAI derivatives did not show any relevant isoform selectivity, but instead exhibit a rather flat inhibition profile against target and off-target isozymes.

### Evaluation of H_2_S Release

**Figure 2:**
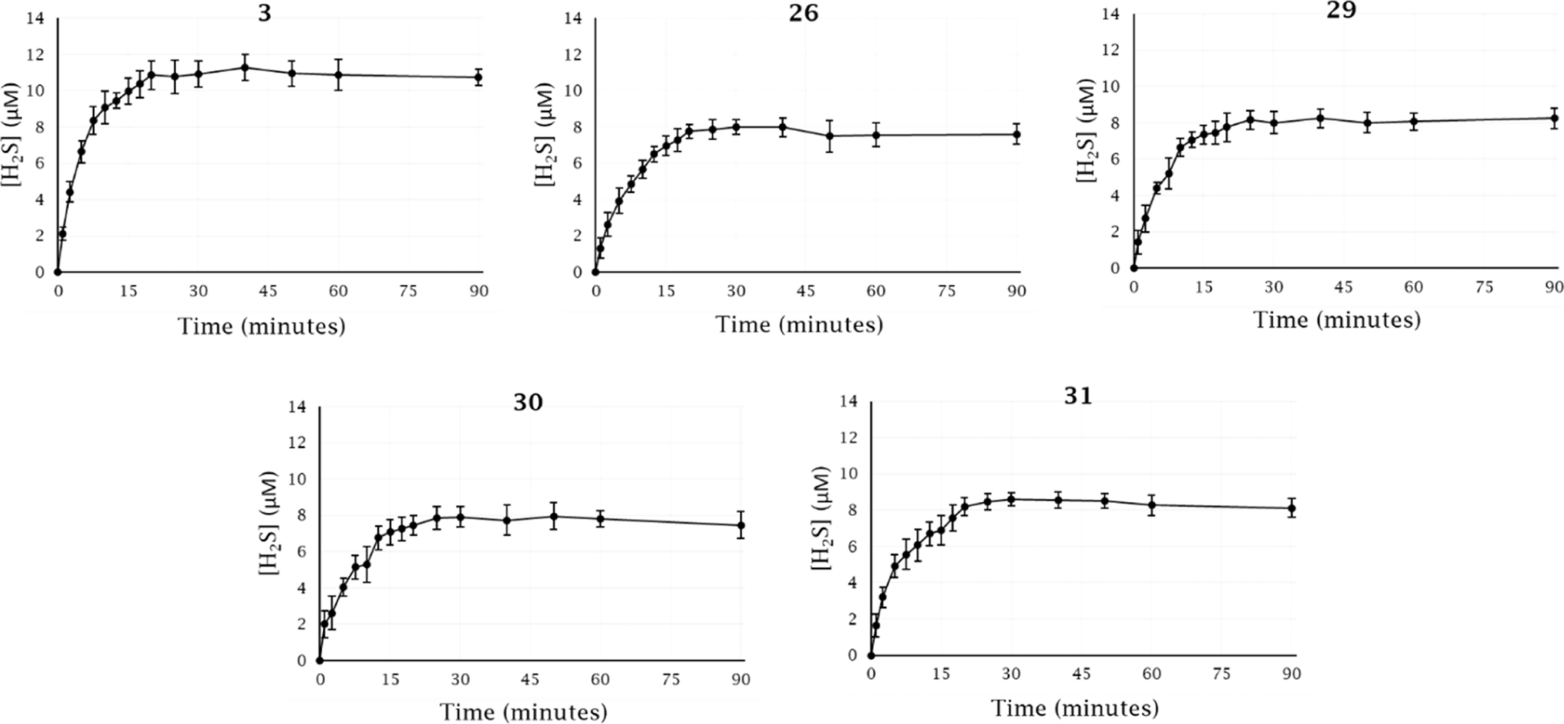
H_2_S releasing profile of compounds **3**, **26**, **29**, **30**, and **31** (150 μM). Compounds were incubated at 37 °C in rat liver homogenate. H_2_S concentration was determined at timed intervals spectrophotometrically. Data are expressed as means ± SEM of three independent assays.

The H_2_S releasing properties of the 4-substituted-3*H*-1,2-dithiole-3-thione precursor **3** and the hybrid derivatives showing the best CA inhibition profiles (in terms of potency and selectivity against the target isoforms), that are **26**, **29**, **30**, and **31**, were evaluated by a spectrophotometric methylene blue (MB^+^) assay adapted from previously reported methods.^40,41^ The released H_2_S, trapped by zinc acetate to form zinc sulfide, reacts with *N*,*N*-dimethyl-1,4-phenylenediamine in the presence of iron(III) to yield methylene blue, which can be easily quantified by measuring absorbance at 667 nm (MB^+^ form) or at 748 nm (MBH^2+^).^42,43^ The compound H_2_S release in different conditions was monitored over time and quantified according to a calibration curve with Na_2_S (1–300 μM). Incubation of compound **3** and the hybrid derivatives in phosphate buffer at 37 °C either in the presence or absence of thiol compounds (cysteine or glutathione) produced no or a barely detectable H_2_S release, respectively. Therefore, trigger mechanisms other than hydrolysis and activation with thiols were investigated, such as enzymatic/metabolic triggers in rat tissue homogenates as reported in 2011 by Li et al. with similar such compounds.^41^ Incubation of **3, 26**, **29**, **30**, and **31** at 37 °C in rat liver homogenate resulted in an almost immediate and sustained release of H_2_S over the full 90 min incubation period, implying that the most released gas from the molecules occurred as a result of a metabolic event. At present, the nature of this activation step is not known. Table S3, Supporting Information lists the parameters of *C*_max_ (the highest concentration of H_2_S released) and *t*_1/2_ (the time at which half the *C*_max_ is reached) from the tested compounds. The parent compound **3** showed the highest H_2_S release in terms of the rate and *C*_max_. Its H_2_S release peaked at a concentration of 12.1 μM after 20 min approximately and remained as such with slight fluctuations for the residual analysis time (Figure 2), with a *t*_1/2_ of 3.1 min.

In comparison to precursor **3**, the tertiary amide derivatives **26**, **29**, **30**, and **31** exhibited a weaker H_2_S release (Figure 2), with an overall superimposable trend for all compounds, implying that the amide substituents barely affect the compound’s ability to release H_2_S. The latter seems instead in part affected by the carboxylic acid (**3**) to amide (**26**, **29**, **30**, and **31**) conversion. The H_2_S *C*_max_ values of the amide derivatives settle in the range of 7.9–8.4 μM, with *t*_1/2_ in the range of 4.3–5.0 min (Table S3, Supporting Information). The gasotransmitter release of the tested hybrid compounds overall reached a plateau after approximately 25 min. The H_2_S release profile of the DTT derivatives here tested is consistent with that of previously described similar such derivatives, although differences exist in the compound chemical structures and in the adopted spectrophotometric protocol. As a matter of fact, in 2007 Li et al. showed in vitro consistent H_2_S release profiles of the 5-substituted **DTT** compound ADTOH and its diclofenac derivative, when incubated in rat liver homogenate.^41^ The same authors evaluated successively ADTOH and the 4-substituted analogue ACS48 (compound **3** in the present work) for their H_2_S donating properties in the cell, also showing a sustained release.^44^ In 2017, Li et al. also evaluated, in vitro, a similar H_2_S release for ADTOH and some such nonsteroidal anti-inflammatory derivatives.^45^ The authors deduced a hydrolytic trigger for the compound gasotransmitter release, but the reducing agent TECP, that is tris(2-carboxyethyl)phosphine, was actually used. In 2018 Giustarini et al. also demonstrated the sustained H_2_S release of **3** (ACS48), in vivo, by monitoring the gasotransmitter concentration in plasma of rats treated intravenously with such a DTT and some of its derivatives.^35^ As a result, to the best of our knowledge, the 4-substituted DDT **3** and amide derivatives, such as **19–33**, were here assessed for the first time for their H_2_S release, in vitro.

### In Vivo Studies

On the basis of the superimposable H_2_S releasing profiles of all tested hybrid derivatives, **26**, **29**, **30**, and **31** were also evaluated for their anti-inflammatory effect in a rat model of arthritis induced by the intra-articular injection of complete Freund’s adjuvant (CFA) in the Paw pressure and Incapacitance tests.^33^ Moreover, the synthetic precursors **3**, as the single H_2_S releasing molecule, and **15**, as the most potent single CAI warhead against the target hCAs IX and XII, were tested to evaluate the hybrid efficacy with respect to the single agents, and their coadministration. The CFA model induces pain and articular damage characterized by diffuse lesions caused by an immune reaction towards antigens of connective tissue or joint.^46^ The primary target of this response is the collagen that can be disrupted by T cells activation. The pathogenesis is characterized by augmented levels of pro-inflammatory cytokines,^47^ neutrophil infiltration and subsequent synovia hyperplasia with a total ablation of the joint space that is replaced by hyperplastic tissue.^48^

**Figure 3:**
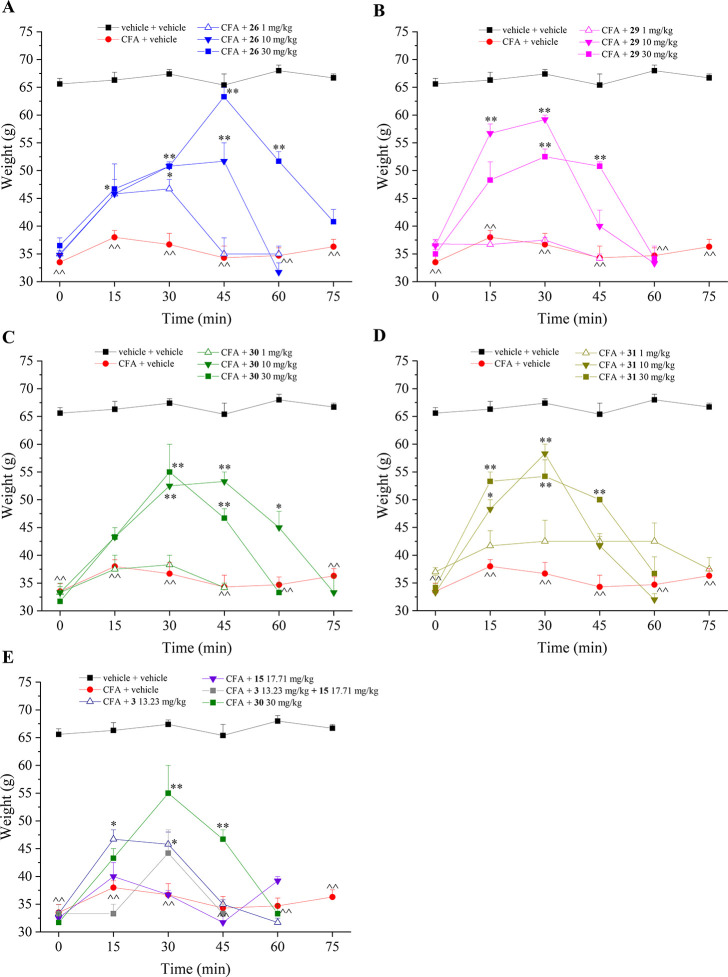
Acute pain-relieving effect of hybrid **26**, **29–31** (A–D) H_2_S donor **3** and CAI **15** (E) in an adjuvant-induced arthritis model. The compounds were administered in a range of doses from 1 to 30 mg/kg, while 3 and 15 were administered at the equimolar dose of 30 mg/kg of hybrid derivative **30** (13.23 and 17.71, respectively). Compounds were suspended in 1% CMC and orally administered. The measurements of mechanical hyperalgesia were accomplished on day 14 after CFA injection by the Paw pressure test. The values reported are the mean of 8 rats performed in 2 different experimental sets. ^̂*P* < 0.01 vs vehicle + vehicle-treated animals; *P < 0.05 and **P < 0.01 vs CFA + vehicle group.

The pain-relieving properties of compounds were evaluated after a single per *os* administration in a dose ranging from 1 to 30 mg/kg when pain and articular lesions were well established (day 14 from the induction of the damage). The Paw pressure test was used to measure the hypersensitivity in response to a mechanical noxious stimulus (Figure 3). In the group treated with CFA (red line), the weight tolerated on the ipsilateral paw decreased to 33 g in comparison to the control value of 65 g (black line) in the untreated rats (vehicle + vehicle). Compound **26** evoked an antihypersensitivity effect in a dose-dependent manner. In particular, the dose of 30 mg/kg counteracted the mechanical hyperalgesia 45 min after injection, showing a mild effect also at 60 min. The lower doses were still active and even showed a lower efficacy (Figure 3A). Compounds **29**, **30**, and **31** were active in reducing the mechanical hyperalgesia evoked by CFA intra-articular (i.a.) injection with no dose-response effect between the dose of 10 and 30 mg/kg. Both dosages significantly increased the weight tolerated by the animals on the ipsilateral paw in comparison to CFA-treated animals starting from 15 min up to 45 min after treatment. For all these three hybrids, the lower dose of 1 mg/kg was ineffective (Figure 3B–D). Figure 3E reported the comparison between the antihyperalgesic effect evoked by hybrid **30** 30 mg/kg in comparison to the equimolar doses of its single portions, that is the H_2_S donor **3** and the CAI **15**, selected for being the most potent inhibitor of hCAs IX and XII among the secondary amine derivatives **4–18**. The effect of **30** was also compared to the co-administration of **3** and **15**. Despite **30** not standing as the best hybrid compound in terms of in vivo efficacy in the Paw pressure test, it clearly showed a greater antihyperalgesic action with respect to **3**, **15**, and their co-administration, highlighting the advantage of our strategy intended to merge in a single-molecular entity the two therapeutically active portions (Figure 3E). Unlike the hybrid derivatives, the single H_2_S donor **3** evoked a rapid antihyperalgesic effect that peaked at 15 min and totally disappeared after 45 min. Such a trend might be partially justified by the greater H_2_S release rate detected for compound **3** (Figure 2) in comparison to the other amide derivatives. Interestingly, compound **15**, which possesses CA inhibition properties only, showed a very weak antihyperalgesic action at the dose here evaluated (17.71 mg/kg) equimolar to the active dose of the hybrid compounds (30 mg/kg). As a matter of fact, in the previous study regarding CO releaser—CAI hybrid compounds, a benzenesulfonamide CAI derivative inducing a potent inhibition of the target hCAs IX and XII, referred to as **3a**,^30^ showed a detectable pharmacological action in the Paw pressure test only when administered at a 4-fold higher molar dose than **15**. The different dosages administered for **15** (present study) and **3a** (past study)^30^ depend on the 4-fold lower dose necessary for the best hybrid H_2_S releaser here reported (**26**) to achieve a pharmacological action even higher than the best hybrid CAI—CO releaser (referred as **5b**)^30^ in the Paw pressure test. In contrast to the rapid action of the H_2_S donor **3**, the CAI **3a** exhibited a peak of antihyperalgesic at 45 min.^30^

To exclude possible toxicity induced by the acute treatment with the described hybrids, autonomic, behavioral, and neurological parameters were assessed by the Irwin test. As reported in Table S4, Supporting Information the highest dose of all compounds did not alter the endpoints analyzed.

**Figure 4:**
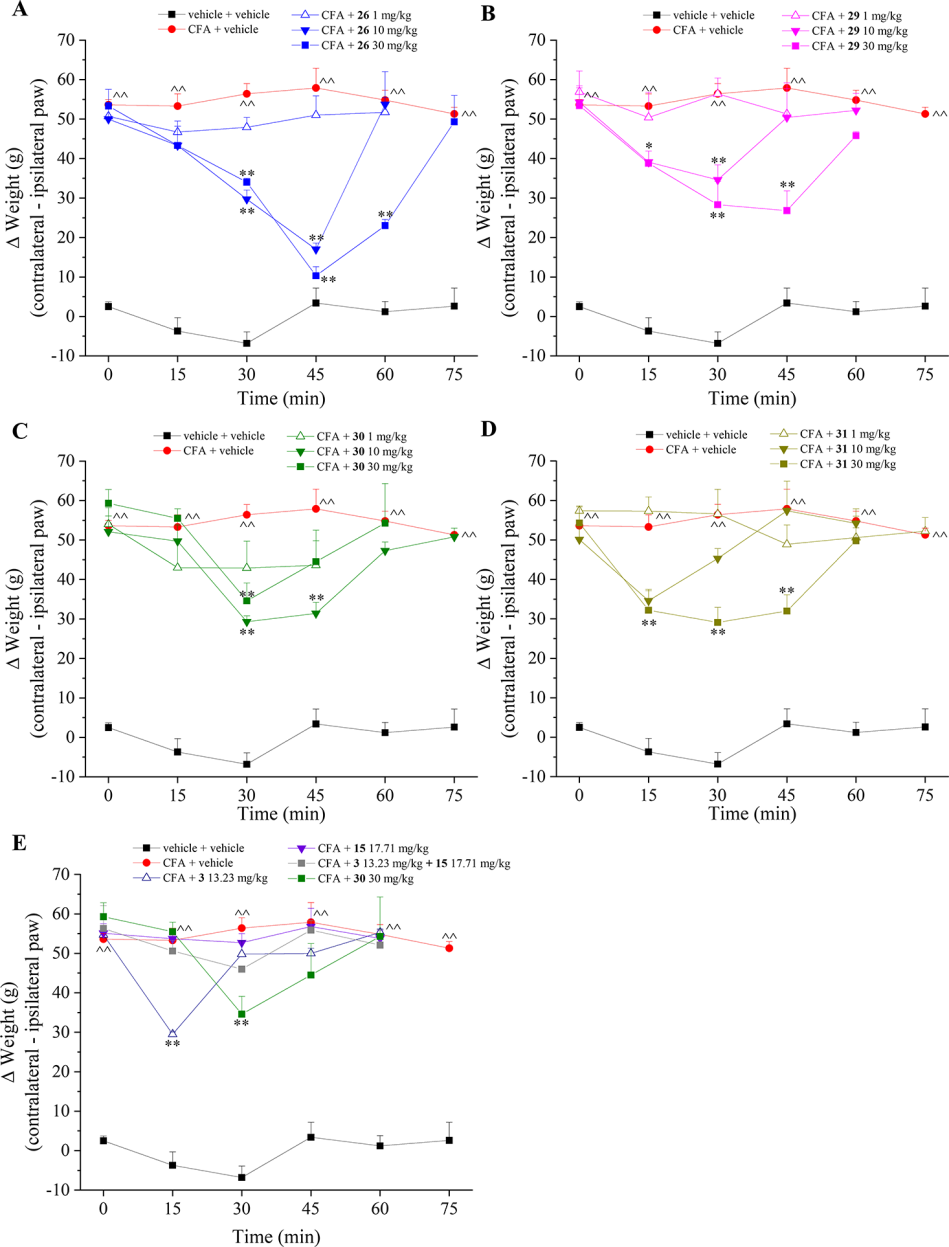
Acute pain-relieving effect of the hybrid compounds **26**, **29–31** (A–D) H_2_S donor **3** and CAI **15** (E) in an adjuvant-induced arthritis model. Hybrids were administered in a range of doses from 1 to 30 mg/kg, while **3** and **15** were administered at the equimolar dose of 30 mg/kg of hybrid **30** (13.23 and 17.71, respectively). Compounds were suspended in 1% CMC and orally administered. The measurements of spontaneous pain were accomplished on day 14 after CFA injection by the Incapacitance test. The values reported are the mean of 8 rats performed in 2 different experimental sets. ^̂*P* < 0.01 vs vehicle + vehicle-treated animals; *P < 0.05 and **P < 0.01 vs CFA + vehicle group.

The hybrid derivatives **26**, **29**, **30**, and **31** were also tested against postural unbalance, a typical feature of monolateral pain, using the Incapacitance test as an evaluation of spontaneous pain since no direct noxious or non-noxious stimuli are applied by the operator (Figure 4). The difference between the weight bore on the contralateral and the ipsilateral paw was significantly increased up to 55 g (red line) in CFA-treated rats in comparison to the 2 g of the control group (black line). As in the Paw pressure test, similar results were obtained in this behavioral measurement. Compounds **26**, **29**, and **31** reduced spontaneous pain in a dose-dependent manner with a peak of efficacy between 30 and 45 min after treatment (Figure 4A,B,D). Again, compound **26** demonstrated the greatest antihyperalgesic action almost completely reverting spontaneous pain after 45 min. Compound **30** oddly showed a slightly greater efficacy at 10 mg/kg in comparison to the dose of 30 mg/kg, while the lowest dose was ineffective (Figure 4C). In the Incapacitance test, compound **30** induced a greater antihyperalgesic action than the co-administration of **3** and **15** (Figure 4E). Nonetheless, in this test the action of compound **3** alone also exceeded that of the combination therapy and was comparable to that of the hybrid analogue **30** at its optimal dose (Figure 4E). Again, the antihyperalgesic effect of the H_2_S releaser **3** rapidly peaked at 15 min, already disappearing after 30 min. Again, in absence of effects detected in vivo for **15** at the used dose, the CAI **3a** previously reported^30^ can be used to prove the antihyperalgesic action of a single CAI warhead in the Incapacitance test. In the latter, compound **3a**, tested at a 4-fold greater dose than **15**, exhibited a weak action with a peak at 45 min.

Relevantly, also in the Incapacitance test, the antihyperalgesic action of the best H_2_S releaser hybrid **26** significantly exceeded, according to a 4-fold lower administered dose (30 vs 100 mg/kg, and greater molecular weight), that induced by the best hybrid CAI—CO releaser (**5b**), which in turn was comparable to the reference drug ibuprofen 100 mg/kg.^30^

In both in vivo tests, the antihyperalgesic action of the evaluated hybrid derivatives reached a maximum effect after 30 min post-administration with the exception of compound **26**, which showed better efficacy and prolonged action over analogues **29–31**. The H_2_S release of the hybrid derivatives, detected by a methylene blue assay in a sealed system, showed a plateau after 25 min approximately. In contrast, a more sustained H_2_S release was notified in vivo (open system) for similar such derivatives,^41^ which might account for an anti-inflammatory action peak in the range 30–45 min. On the other hand, it can be supposed that the trend of the hybrid in vivo action is consistent with a biological response being the result of the merged H_2_S release (peak at 15 min) and target CA inhibition (peak at 45 min as previously shown).^30^

The detected enzymatic/metabolic H_2_S release trigger for the DTT scaffold, rather than hydrolysis and thiol activation, might be an advantage for the setup of such an anti-inflammatory therapy. In fact, it can be supposed that the DTT derivatives start the gasotransmitter release only after absorption has occurred.

On the basis of the multitarget or hybridization approach here proposed, the exceptionally improved antihyperalgesic profile of the hybrid derivatives over the single agents and their co-administration could be rationalized as upgraded pharmacokinetics driving both pharmacophores (H_2_S donor and CAI), combined as a unique molecule, to the target inflamed site, where a strong synergistic effect takes place. Additionally, it can be speculated that the pharmacophore hybridization enhances drug absorption, which may be instead diminished in case of a coadministration.

## Conclusions

H_2_S is a ubiquitous small gaseous signaling molecule, playing an important role in many physio-pathological processes. For instance, H_2_S-donating molecules attracted enormous interest as they were able to promote the resolution of inflammation at the low endogenous gas concentration. On the other hand, some of us recently showed that a panel of CA isoforms, with CAs IX and XII as the main targets, are implicated in the pathogenesis of inflammation, and their inhibitors can repristinate the normal pH in the acidic inflamed synovial fluid, alleviating arthritis symptoms.

Hence, we described herein for the first time the synthesis and biological evaluation of new hybrid derivatives possessing a CAI warhead of the sulfonamide type conjugated to a DTT core as an H_2_S donating molecule. All hybrid compounds were tested in vitro as inhibitors of human (h) CA I, II, IV, IX, and XII, showing a marked increase in isoform-selectivity upon the proposed hybridization strategy over the CAI synthetic precursors. Compounds **26** and **31** were the most potent and selective hCA IX inhibitors with single-digit *K*_I_s of 4.1 and 2.4 nM, respectively, and hCA II/hCA IX SI of 1034 and 719, respectively. Also, **26** and **31** were among the best hCA XII inhibitors with *K*_I_s of 7.7 and 8.0 nM, respectively, and hCA II/hCA XII SI of 383 and 299, respectively. The best among such compounds, **26**, **29**, **30**, and **31**, along with the precursor and previously reported DTT **3**, were shown to consistently release H_2_S by a spectrophotometric methylene blue method. Compounds **26**, **29**, **30**, and **31** induced a pain-relieving effect in a rat model adjuvant-induced arthritis with a single i.a. injection of CFA. Compound **26** showed the greatest efficacy in vivo completely reverting pain 45 min after treatment both in the Paw pressure and Incapacitance tests. All tested compounds demonstrated a markedly greater antihyperalgesic action compared to treatment with the single H_2_S donor **3**, the CAI fragment **15**, and their co-administration. A comparison was thus made with previously reported CAI—CO releaser hybrid derivatives.^30^ Relevantly, the performance of the best H_2_S donor hybrid **26** significantly exceeded, according to a 4-fold lower administered dose (30 vs 100 mg/kg), that induced by the best hybrid CAI—CO releaser (**5b**), which in turn was comparable to the reference drug ibuprofen 100 mg/kg. Compound toxicity induced by the acute treatment was excluded from evaluating the animal autonomic, behavioral, and neurological parameters by the Irwin test. In conclusion, the hybridization drug design approach here proposed drastically improved the anti-inflammatory profile of the hybrid derivatives over the single agents and their coadministration and provided new outstanding biomedical tools for the development of new potent anti-inflammatory treatment against arthritis based on H_2_S release and CA inhibition.

## Experimental Section

### Chemistry

Anhydrous solvents and all reagents were purchased from Sigma-Aldrich, Alfa Aesar, and TCI. All reactions involving air- or moisture-sensitive compounds were performed under a nitrogen atmosphere using dried glassware and syringes techniques to transfer solutions. Nuclear magnetic resonance (^1^H NMR, ^13^C NMR, ^19^F NMR) spectra were recorded using a Bruker Advance III 400 MHz spectrometer in dimethylsulfoxide (DMSO)-*d*_6_. Chemical shifts are reported in parts per million (ppm), and the coupling constants (*J*) are expressed in hertz (Hz). Splitting patterns are designated as follows: s, singlet; d, doublet; sept, septet; t, triplet; q, quadruplet; m, multiplet; brs, broad singlet; and dd, double of doublets. The assignment of exchangeable protons (O*H* and N*H*) was confirmed by the addition of D_2_O. Analytical thin-layer chromatography (TLC) was carried out on Sigma-Aldrich silica gel F-254 plates. Flash chromatography purifications were performed on Sigma-Aldrich Silica gel 60 (230–400 mesh ASTM) as the stationary phase and ethyl acetate (EtOAc)/*n*-hexane or MeOH/dichloromethane (DCM) were used as eluents. Melting points (mp) were measured in open capillary tubes with a Gallenkamp MPD350.BM3.5 apparatus and are uncorrected. All compounds were >95% pure by HPLC analysis, performed by using a Waters 2690 separation module coupled with a photodiode array detector (PDA Waters 996) and as column a Nova-Pak C18 4 μm 3.9 mm × 150 mm (Waters) silica-based reverse phase column. The sample was dissolved in acetonitrile 10%, and an injection volume of 45 μL was used. The mobile phase, at a flow rate of 1 mL/min, was a gradient of water + trifluoroacetic acid (TFA) 0.1% (A) and acetonitrile + TFA 0.1% (B), with steps as follows: (A%:B%), 0–10 min 90:10, 10–25 min gradient to 60:40, 26–28 min isocratic 20:80, and 29–35 min isocratic 90:10. TFA 0.1% in water as well in acetonitrile was used as the counterion.

#### Synthesis of Ethyl 4-Isopropylbenzoate (**1**)

Thionyl chloride (1.8 equiv) was added dropwise to a solution of 4-isopropylbenzoic acid (12.7 mmol, 1.0 equiv) in EtOH (30 mL) cooled at 0 °C, and the reaction mixture was stirred to 60 °C for 6 h. The solvent was evaporated under a vacuum, and the crude was dissolved in EtOAc (40 mL) and washed with water and NaHCO_3_ sat. sol. (30 mL × 2). The organic layer was dried over Na_2_SO_4_, filtered, and evaporated under a vacuum. The obtained colorless oil was purified with flash chromatography (EtOAc 20%/Hex) to give **1**. Yield 92%; δ_H_ (400 MHz, DMSO-*d*_6_): 7.78 (d, *J* = 8.1 Hz, 2H, Ar–*H*), 7.29 (d, *J* = 8.2 Hz, 2H, Ar–*H*), 4.19 (q, *J* = 7.1 Hz, 2H, C*H*_2_), 2.86 (hept, *J* = 6.9 Hz, 1H, C*H*), 1.21 (t, *J* = 7.1 Hz, 3H, CH_2_C*H*_3_), 1.11 (d, *J* = 6.9 Hz, 6H, CH(C*H*_3_)_2_). *m*/*z* (ESI positive) 192.1 [M + H]^+^.

#### Synthesis of Ethyl 4-(3-Thioxo-3*H*-1,2-dithiol-4-yl)benzoate (**2**)

Compound **1** (10.4 mmol, 1.0 equiv) was added dropwise to stirred melted sulfur (10 equiv) at 135 °C, and the reaction mixture was stirred at 220 °C for 6–8 h. The temperature is lowered to 135 °C, and after the addition of 20 mL of a toluene/acetone mixture (3:7), the suspension was triturated at r.t. The solvent was evaporated under vacuum, and the residue was purified with flash chromatography (EtOAc 20%/Hex) to give **2** as a brown powder. Yield 75%; δ_H_ (400 MHz, DMSO-*d*_6_): 9.26 (s, 1H, =C*H*), 8.02 (d, *J* = 8.5 Hz, 2H, Ar–*H*), 7.73 (d, *J* = 8.5 Hz, 2H, Ar–*H*), 4.34 (q, *J* = 7.1 Hz, 2H, C*H*_2_), 1.33 (t, *J* = 7.1 Hz, 3H, C*H*_3_). *m*/*z* (ESI positive) 281.9 [M + H]^+^.

#### Synthesis of 4-(3-Thioxo-3*H*-1,2-dithiol-4-yl)benzoic Acid (**3**)

H_2_SO_4_ 9 M (16 mL) was added to a solution of **2** (7.09 mmol, 1.0 equiv) in acetic acid (50 mL), and the reaction mixture was stirred at 100 °C for 4 h. After cooling at r.t. and adding slush, the mixture was extracted with MeOH 10%/DCM (40 mL × 3) and washed with HCl 0.1 M (1 × 30 mL). The organic layer was dried over Na_2_SO_4_, filtered, and evaporated under a vacuum. The obtained residue was triturated with diethyl ether, collected by filtration, and purified with flash chromatography (EtOAc 50%/Hex) to give **3** as a brown-orange powder. Yield 81%; mp 235–236 °C; δ_H_ (400 MHz, DMSO-*d*_6_): 12.94 (bs, 1H, exchange with D_2_O, COO*H*), 9.25 (s, 1H, =C*H*), 8.00 (d, *J* = 8.3 Hz, 2H, Ar–*H*), 7.71 (d, *J* = 8.2 Hz, 2H, Ar–*H*). *m*/*z* (ESI positive) 253.9 [M + H]^+^.

### General Synthetic Procedures for Secondary Amines **4–18**

#### Procedure 1

The appropriate aldehyde (1.1 equiv) was added to a solution of 4-(2-aminoethyl)benzenesulfonamide (4,99 mmol, 1.0 equiv) in dry MeOH (20 mL), and the mixture was heated under stirring to reflux temperature for 0.5–4 h. Sodium borohydride (1.6 equiv) was added portion-wise at 0 °C, and the reaction mixture was heated under stirring to reflux temperature for 0.5–3 h. The solvent was evaporated under a vacuum, water was added (25 mL) to it, and it was neutralized with HCl 1 M. The suspension was filtered, and the collected powder was occasionally purified with flash chromatography (MeOH 5%/DCM) to give the compounds **4–16**.

#### Procedure 2

Triethylamine (1.2 equiv) and the appropriate halide (1.1 equiv) were added to a solution of 4-(2-aminoethyl)benzenesulfonamide (4,99 mmol, 1.0 equiv) in dry DMF (3 mL) at r.t. and the mixture was stirred at 60 °C for 8 h (**17**) or r.t. for 0.5 h (**18**). The reaction mixture was quenched by the addition of water (10 mL) and extracted with DCM (20 mL × 3). The organic layer was collected, washed with brine (30 mL × 3), dried over Na_2_SO_4_, filtered, and evaporated under vacuum to give a yellow (**17**) or white (**18**) powder.

#### 4-(2-(Benzylamino)ethyl)benzenesulfonamide (**4**)

Compound **4** was obtained as white powder according to the general procedure 1 reported earlier using benzaldehyde (1.1 equiv). Yield 96%; mp 173–175 °C; silica gel TLC *R*_*f*_ 0.08 (TFA/MeOH/DCM 3/5/92% v/v). δ_H_ (400 MHz, DMSO-*d*_6_): 7.76 (d, *J* = 8.1 Hz, 2H, Ar–*H*), 7.42 (m, 7H, Ar–*H*), 7.32 (s, 2H, exchange with D_2_O, SO_2_N*H*_2,_ overlap with signal at 7.42), 4.04 (s, 2H, C*H*_2_), 3.07 (m, 2H, C*H*_2_), 2.97 (m, 2H, C*H*_2_). δ_C_ (100 MHz, DMSO-*d*_6_): 145.87, 142.74, 141.80, 129.99, 129.05, 128.86, 127.46, 126.55, 53.77, 50.91, 36.50. *m*/*z* (ESI positive) 291.1.

#### 4-(2-((4-Fluorobenzyl)amino)ethyl)benzenesulfonamide (**5**)

Compound **5** was obtained as a white powder according to the general procedure 1 reported earlier using 4-fluorobenzaldehyde (1.1 equiv). Yield 95%; silica gel TLC *R*_*f*_ 0.21 (TFA/MeOH/DCM 3/5/92% v/v). δ_H_ (400 MHz, DMSO-*d*_6_): 7.73 (d, *J* = 8.2 Hz, 2H, Ar–*H*), 7.38 (m, 4H, Ar–*H*), 7.28 (s, 2H, exchange with D_2_O, SO_2_N*H*_2,_ overlap with signal at 7.38), 7.12 (t, *J* = 8.8 Hz, 2H, Ar–*H*), 3.73 (s, 2H, C*H*_2_), 2.79 (m, 4H, 2 x C*H*_2_). δ_F_ (376 MHz, DMSO-*d*_6_): −116.18. δ_C_ (100 MHz, DMSO-*d*_6_): 145.61, 142.94, 131.06, 130.98, 130.10, 126.73, 115.96, 115.75, 52.74, 50.62, 36.18. *m*/*z* (ESI positive) 309.1 [M + H]^+^.

#### 4-(2-((4-Chlorobenzyl)amino)ethyl)benzenesulfonamide (**6**)

Compound **6** was obtained as a white powder according to the general procedure 1 reported earlier using 4-chlorobenzaldehyde (1.1 equiv). Yield 87%; silica gel TLC *R*_*f*_ 0.35 (TFA/MeOH/DCM 3/5/92% v/v). δ_H_ (400 MHz, DMSO-*d*_6_): 7.73 (d, *J* = 7.7 Hz, 2H, Ar–*H*), 7.39 (d, *J* = 7.9 Hz, 2H, Ar–*H*), 7.36 (s, 4H, Ar–*H*), 7.28 (s, 2H, exchange with D_2_O, SO_2_N*H*_2_), 3.76 (s, 2H, C*H*_2_), 2.80 (m, 4H, 2 × C*H*_2_). δ_C_ (100 MHz, DMSO-*d*_6_): 145.30, 142.84, 139.65, 132.31, 130.96, 129.99, 129.03, 126.60, 52.47, 50.38, 35.87. *m*/*z* (ESI positive) 325.1 [M + H]^+^.

#### 4-(2-((4-Bromobenzyl)amino)ethyl)benzenesulfonamide (**7**)

Compound **7** was obtained as a white powder according to the general procedure 1 reported earlier using 4-bromobenzaldehyde (1.1 equiv). Yield 85%; silica gel TLC *R*_*f*_ 0.38 (TFA/MeOH/DCM 3/5/92% v/v). δ_H_ (400 MHz, DMSO-*d*_6_): 7.80 (d, *J* = 8.1 Hz, 2H, Ar–*H*), 7.62 (d, *J* = 8.2 Hz, 2H, Ar–*H*), 7.47 (m, 4H, Ar–*H*), 7.37 (s, 2H, exchange with D_2_O, SO_2_N*H*_2_), 4.02 (s, 2H, C*H*_2_), 3.03 (s, 4H, 2 × C*H*_2_). δ_C_ (100 MHz, DMSO-*d*_6_): 143.70, 143.38, 135.48, 132.73, 132.50, 130.30, 127.00, 122.54, 51.41, 49.24, 33.77. *m*/*z* (ESI positive) 369.0 [M + H]^+^.

#### 4-(2-((4-Cyanobenzyl)amino)ethyl)benzenesulfonamide (**8**)

Compound **8** was obtained as a white powder according to the general procedure 1 reported earlier using 4-cyanobenzaldehyde (1.1 equiv). Yield 91%; silica gel TLC *R*_*f*_ 0.31 (TFA/MeOH/DCM 3/5/92% v/v). δ_H_ (400 MHz, DMSO-*d*_6_): 7.75 (d, *J* = 8.1 Hz, 2H, Ar–*H*), 7.71 (d, *J* = 8.1 Hz, 2H, Ar–*H*), 7.49 (d, *J* = 8.0 Hz, 2H, Ar–*H*), 7.37 (d, *J* = 8.2 Hz, 2H, Ar–*H*), 7.29 (s, 2H, exchange with D_2_O, SO_2_N*H*_2_), 3.79 (s, 2H, C*H*_2_), 2.76 (m, 4H, 2 x C*H*_2_), 2.32 (bs, 1H, exchange with D_2_O, N*H*). δ_C_ (100 MHz, DMSO-*d*_6_): 148.09, 145.61, 143.17, 133.06, 130.03, 129.75, 126.60, 120.08, 110.25, 53.24, 50.93, 36.54. *m*/*z* (ESI positive) 316.1 [M + H]^+^.

#### 4-(2-((4-(Dimethylamino)benzyl)amino)ethyl)benzenesulfonamide (**9**)

Compound **9** was obtained as a white powder according to the general procedure 1 reported earlier using 4-(dimethylamino)benzaldehyde (1.1 equiv). Yield 78%; silica gel TLC *R*_*f*_ 0.24 (TFA/MeOH/DCM 3/5/92% v/v). δ_H_ (400 MHz, DMSO-*d*_6_): 7.76 (d, *J* = 8.2 Hz, 2H, Ar–*H*), 7.42 (d, *J* = 8.2 Hz, 2H, Ar–*H*), 7.35 (s, 2H, exchange with D_2_O, SO_2_N*H*_2_), 7.28 (d, *J* = 8.5 Hz, 2H, Ar–*H*), 6.70 (d, *J* = 8.6 Hz, 2H, Ar–*H*), 3.91 (s, 2H, C*H*_2_), 2.99 (s, 4H, 2 × C*H*_2_), 2.88 (s, 6H, 2 x C*H*_3_). δ_C_ (100 MHz, DMSO-*d*_6_): 151.33, 143.40, 143.26, 131.57, 130.04, 126.86, 121.66, 112.98, 51.26, 48.22, 40.99, 33.06. *m*/*z* (ESI positive) 334.2 [M + H]^+^.

#### 4-(2-((4-Nitrobenzyl)amino)ethyl)benzenesulfonamide (**10**)

Compound **10** was obtained as a yellow powder according to the general procedure 1 reported earlier using 4-nitrobenzaldehyde (1.1 equiv). Yield 94%; silica gel TLC *R*_*f*_ 0.17 (TFA/MeOH/DCM 3/5/92% v/v). δ_H_ (400 MHz, DMSO-*d*_6_): 8.16 (d, *J* = 8.6 Hz, 2H, Ar–*H*), 7.72 (d, *J* = 8.2 Hz, 2H, Ar–*H*), 7.57 (d, *J* = 8.6 Hz, 2H, Ar–*H*), 7.39 (d, *J* = 8.2 Hz, 2H, Ar–*H*), 7.26 (s, 2H, exchange with D_2_O, SO_2_N*H*_2_), 3.84 (s, 2H, C*H*_2_), 2.82 (m, 2H, C*H*_2_), 2.73 (m, 2H, C*H*_2_), 2.40 (bs, 1H, exchange with D_2_O, N*H*). δ_C_ (100 MHz, DMSO-*d*_6_): 150.49, 147.34, 145.84, 142.88, 130.12, 129.84, 126.69, 124.29, 53.05, 51.00, 36.63. *m*/*z* (ESI positive) 336.1 [M + H]^+^.

#### 4-(2-((2-Methoxy-4-nitrobenzyl)amino)ethyl)benzenesulfonamide (**11**)

Compound **11** was obtained as a white powder according to the general procedure 1 reported earlier using 2-methoxy-4-nitrobenzaldehyde (1.1 equiv). Yield 87%; silica gel TLC *R*_*f*_ 0.15 (TFA/MeOH/DCM 3/5/92% v/v). δ_H_ (400 MHz, DMSO-*d*_6_): 7.83 (dd, *J* = 8.3, 2.1 Hz, 1H, Ar–*H*), 7.73 (m, 3H, Ar–*H*), 7.59 (d, *J* = 8.3 Hz, 1H, Ar–*H*), 7.41 (d, *J* = 8.3 Hz, 2H, Ar–*H*), 7.30 (s, 2H, exchange with D_2_O, SO_2_N*H*_2_), 3.90 (s, 3H, C*H*_3_), 3.86 (s, 2H, C*H*_2_), 2.85 (s, 4H, 2 × C*H*_2_). δ_C_ (100 MHz, DMSO-*d*_6_): 158.31, 148.56, 145.02, 142.96, 136.08, 130.50, 130.06, 126.66, 116.32, 106.04, 57.04, 50.48, 47.42, 35.60. *m*/*z* (ESI positive) 366.1 [M + H]^+^.

#### 4-(2-((4-Methoxybenzyl)amino)ethyl)benzenesulfonamide (**12**)

Compound **12** was obtained as a white powder according to the general procedure 1 reported earlier using 4-methoxybenzaldehyde (1.1 equiv). Yield 86%; silica gel TLC *R*_*f*_ 0.17 (TFA/MeOH/DCM 3/5/92% v/v). δ_H_ (400 MHz, DMSO-*d*_6_): 7.72 (d, *J* = 8.1 Hz, 2H, Ar–*H*), 7.38 (d, *J* = 8.1 Hz, 2H, Ar–*H*), 7.26 (s, 2H, exchange with D_2_O, SO_2_N*H*_2_), 7.21 (d, *J* = 7.6 Hz, 2H, Ar–*H*), 6.85 (d, *J* = 7.6 Hz, 2H, Ar–*H*), 3.72 (s, 3H, OC*H*_3_), 3.64 (s, 2H, C*H*_2_), 2.76 (m, 4H, 2 × C*H*_2_). δ_C_ (100 MHz, DMSO-*d*_6_): 159.02, 145.79, 142.73, 133.41, 130.11, 129.99, 126.57, 114.46, 55.94, 53.08, 50.69, 36.35. *m*/*z* (ESI positive) 321.1 [M + H]^+^.

#### 4-(2-((3,4-Dimethoxybenzyl)amino)ethyl)benzenesulfonamide (**13**)

Compound **13** was obtained as a white powder according to the general procedure 1 reported earlier using 3,4-dimethoxybenzaldehyde (1.1 equiv). Yield 82%; silica gel TLC *R*_*f*_ 0.19 (TFA/MeOH/DCM 3/5/92% v/v). δ_H_ (400 MHz, DMSO-*d*_6_): 7.74 (d, *J* = 8.1 Hz, 2H, Ar–*H*), 7.41 (d, *J* = 8.1 Hz, 2H, Ar–*H*), 7.29 (s, 2H, exchange with D_2_O, SO_2_N*H*_2_), 7.04 (s, 1H, Ar–*H*), 6,89 (s, 2H, Ar–*H*), 3.81 (s, 2H, C*H*_2_), 3.73 (s, 6H, 2 × OC*H*_3_), 2.89 (s, 4H, 2 x C*H*_2_). δ_C_ (100 MHz, DMSO-*d*_6_): 149.65, 149.24, 144.62, 143.08, 130.58, 130.17, 126.80, 122.09, 113.54, 112.57, 56.57, 56.51, 52.52, 49.63, 34.78. *m*/*z* (ESI positive) 351.1 [M + H]^+^.

#### 4-(2-((4-(Methylthio)benzyl)amino)ethyl)benzenesulfonamide (**14**)

Compound **14** was obtained as a white powder according to the general procedure 1 reported earlier using 4-(methylthio)benzaldehyde (1.1 equiv). Yield 88%; silica gel TLC *R*_*f*_ 0.29 (TFA/MeOH/DCM 3/5/92% v/v). δ_H_ (400 MHz, DMSO-*d*_6_): 7.77 (d, *J* = 8.2 Hz, 2H, Ar–*H*), 7.44 (m, 4H, Ar–*H*), 7.33 (s, 2H, exchange with D_2_O, SO_2_N*H*_2_), 7.29 (d, *J* = 8.3 Hz, 2H, Ar–*H*), 4.05 (s, 2H, C*H*_2_), 3.06 (m, 4H, 2 × C*H*_2_), 2.48 (s, 3H, SC*H*_3_). δ_C_ (100 MHz, DMSO-*d*_6_): 143.55, 143.03, 139.97, 131.54, 130.65, 130.27, 127.04, 126.87, 51.09, 48.54, 32.89, 15.64. *m*/*z* (ESI positive) 337.1 [M + H]^+^.

#### 4-(2-((Naphthalen-1-ylmethyl)amino)ethyl)benzenesulfonamide (**15**)

Compound **15** was obtained as a white powder according to the general procedure 1 reported earlier using 4-bromobenzaldehyde (1.1 equiv). Yield 78%; silica gel TLC *R*_*f*_ 0.36 (TFA/MeOH/DCM 3/5/92% v/v). δ_H_ (400 MHz, DMSO-*d*_6_): 8.18 (m, 1H, Ar–*H*), 7.94 (m, 1H, Ar–*H*), 7.84 (d, *J* = 8.0 Hz, 1H, Ar–*H*), 7.77 (d, *J* = 8.1 Hz, 2H, Ar–*H*), 7.52 (m, 6H, Ar–*H*), 7.31 (s, 2H, exchange with D_2_O, SO_2_N*H*_2_), 4.20 (s, 2H, C*H*_2_), 2.89 (s, 4H, 2 × C*H*_2_), 2.13 (bs, 1H, exchange with D_2_O, N*H*). δ_C_ (100 MHz, DMSO-*d*_6_): 145.89, 142.75, 137.29, 134.32, 132.45, 130.03, 129.28, 128.12, 126.76, 126.64, 126.58, 126.52, 126.32, 125.04, 51.56, 51.43, 36.51. *m*/*z* (ESI positive) 341.1 [M + H]^+^.

#### 4-(2-((Benzo[*b*]thiophen-3-ylmethyl)amino)ethyl)benzenesulfonamide (**16**)

Compound **16** was obtained as a white powder according to the general procedure 1 reported earlier using 4-bromobenzaldehyde (1.1 equiv). Yield 83%; silica gel TLC *R*_*f*_ 0.39 (TFA/MeOH/DCM 3/5/92% v/v). δ_H_ (400 MHz, DMSO-*d*_6_): 7.98 (d, *J* = 7.4 Hz, 2H, Ar–*H*), 7.92 (d, *J* = 7.2 Hz, 2H, Ar–*H*), 7.74 (d, *J* = 7.9 Hz, 2H, Ar–*H*), 7.68 (s, 1H, Ar–*H*), 7.40 (m, 4H, Ar–*H*), 7.31 (s, 2H, exchange with D_2_O, SO_2_N*H*_2_), 4.11 (s, 2H, C*H*_2_), 2.94 (m, 4H, 2 x C*H*_2_). δ_C_ (100 MHz, DMSO-*d*_6_): 144.77, 143.00, 140.73, 139.09, 134.05, 130.05, 126.69, 126.03, 125.43, 125.02, 123.79, 123.18, 50.30, 46.48, 35.10. *m*/*z* (ESI positive) 347.1 [M + H]^+^.

#### 4-(2-(Phenethylamino)ethyl)benzenesulfonamide (**17**)

Compound **17** was obtained as a white powder according to the general procedure 2 reported earlier using (2-bromoethyl)benzene (1.1 equiv). Yield 73%; silica gel TLC *R*_*f*_ 0.02 (TFA/MeOH/DCM 3/5/92% v/v). δ_H_ (400 MHz, DMSO-*d*_6_): 7.77 (d, *J* = 8.0 Hz, 2H, Ar–*H*), 7.44 (d, *J* = 8.0 Hz, 2H, Ar–*H*), 7.34 (s, 2H, exchange with D_2_O, SO_2_N*H*_2,_ overlap with signal at 7.44), 7.29 (m, 4H, Ar–*H*), 3.11 (m, 4H, 2 × C*H*_2_), 2.91 (m, 4H, 2 × C*H*_2_). δ_C_ (100 MHz, DMSO-*d*_6_): 145.71, 142.27, 141.02, 130.04, 129.47, 129.26, 126.88, 126.59, 51.35, 50.91, 36.29, 36.05. *m*/*z* (ESI positive) 305.1 [M + H]^+^.

#### 4-(2-((2-Cyanoethyl)amino)ethyl)benzenesulfonamide (**18**)

Compound **18** was obtained as a white powder according to the general procedure 2 reported earlier using 3-chloropropionitrile (1.1 equiv). Yield 85%; silica gel TLC *R*_*f*_ 0.15 (TFA/MeOH/DCM 3/5/92% v/v). δ_H_ (400 MHz, DMSO-*d*_6_): 7.72 (d, *J* = 8.0 Hz, 2H, Ar–*H*), 7.41 (d, *J* = 8.0 Hz, 2H, Ar–*H*), 7.27 (s, 2H, exchange with D_2_O, SO_2_N*H*_2_), 2.76 (m, 6H, 3 x C*H*_2_), 2.57 (t, *J* = 6.6 Hz, 2H, C*H*_2_). δ_C_ (100 MHz, DMSO-*d*_6_): 145.72, 142.88, 130.14, 126.68, 121.19, 50.83, 45.66, 36.59, 18.88. *m*/*z* (ESI positive) 254.0 [M + H]^+^.

### General Synthetic Procedure for the H_2_S Releaser-CAI Hybrids **19–33**

The appropriate secondary amine **4–18** (0.98 equiv), DIPEA (2.2 equiv), and PyBOP (1.1 equiv) were added to a solution of **3** (0.98 mmol, 1.0 equiv) in DMF dry (1 mL), and the reaction mixture was stirred o.n. at r.t. The reaction was quenched with water, and the formed precipitate was collected by filtration and washed with NaHCO_3_ sat. sol. and water. The obtained residue was purified with flash chromatography (MeOH 1%/DCM) to give the compounds **19–33**.

#### *N*-Benzyl-*N*-(4-sulfamoylphenethyl)-4-(3-thioxo-3*H*-1,2-dithiol-4-yl)benzamide (**19**)

Compound **19** was obtained as an orange powder according to the general procedure reported earlier using **4** (1.1 equiv). Yield 72%; mp 139–140 °C. δ_H_ (400 MHz, DMSO-*d*_6_): 9.20 (s, 1H, =C*H*), 7.43 (m, 13H, Ar–*H*), 7.30 (s, 2H, exchange with D_2_O, SO_2_N*H*_2,_ overlap with signal at 7.43), 4.80 (s, 1.1H, C*H*_2_), 4.43 (s, 0.9H, C*H*_2_), 3.59 (m, 1H, C*H*_2_), 3.34 (m, 1H, C*H*_2_), 2.98 (m, 1H, C*H*_2_), 2.85 (m, 1H, C*H*_2_). δ_C_ (100 MHz, DMSO-*d*_6_): 214.15, 214.00, 171.07, 170.97, 160.26, 160.10, 147.79, 147.58, 143.78, 142.85, 142.78, 138.18, 137.30, 136.85, 134.85, 134.67, 129.70, 129.41, 129.25, 129.11, 128.10, 127.67, 127.45, 126.73, 126.58, 126.27, 52.56, 50.19, 47.35, 45.88, 34.18, 32.92. ESI-HRMS (*m*/*z*): [M + H]^+^ calcd for C_25_H_22_N_2_O_3_S_4_, 527.0513; found, 527.0519.

#### *N*-(4-Fluorobenzyl)-*N*-(4-sulfamoylphenethyl)-4-(3-thioxo-3*H*-1,2-dithiol-4-yl)benzamide (**20**)

Compound **20** was obtained as an orange powder according to the general procedure reported earlier using **5** (1.1 equiv). Yield 64%; mp 158–159 °C. δ_H_ (400 MHz, DMSO-*d*_6_): 9.21 (s, 1H, =C*H*), 7.44 (m, 12H, Ar–*H*), 7.29 (s, 2H, exchange with D_2_O, SO_2_N*H*_2,_ overlap with signal at 7.47), 4.77 (s, 1.1H, C*H*_2_), 4.41 (s, 0.9H, C*H*_2_), 3.58 (m, 1H, C*H*_2_), 3.34 (m, 1H, C*H*_2_), 2.98 (m, 1H, C*H*_2_), 2.85 (m, 1H, C*H*_2_). δ_F_ (376 MHz, DMSO-*d*_6_): −115.56 (s, 1F). δ_C_ (100 MHz, DMSO-*d*_6_): 214.16, 214.12, 171.11, 170.94, 160.21, 160.11, 148.62, 147.73, 147.57, 146.90, 144.76, 143.76, 142.79, 141.22, 139.78, 138.15, 136.76, 134.71, 134.43, 130.17, 130.04, 129.70, 129.41, 126.69, 126.60, 126.26, 51.85, 50.14, 46.69, 45.87, 34.19, 32.96. ESI-HRMS (*m*/*z*): [M + H]^+^ calcd for C_25_H_21_FN_2_O_3_S_4_, 545.0419; found, 545.0424.

#### *N*-(4-Chlorobenzyl)-*N*-(4-sulfamoylphenethyl)-4-(3-thioxo-3*H*-1,2-dithiol-4-yl)benzamide (**21**)

Compound **21** was obtained as an orange powder according to the general procedure reported earlier using **6** (1.1 equiv). Yield 52%; mp 104–105 °C. δ_H_ (400 MHz, DMSO-*d*_6_): 9.21 (s, 1H, =C*H*), 7.44 (m, 12H, Ar–*H*), 7.30 (s, 2H, exchange with D_2_O, SO_2_N*H*_2,_ overlap with signal at 7.47), 4.76 (s, 1.1H, C*H*_2_), 4.40 (s, 0.9H, C*H*_2_), 3.57 (m, 1H, C*H*_2_), 3.34 (m, 1H, C*H*_2_), 2.98 (m, 1H, C*H*_2_), 2.85 (m, 1H, C*H*_2_). δ_C_ (100 MHz, DMSO-*d*_6_): 214.04, 213.96, 171.18, 160.02, 147.81, 147.55, 143.73, 142.80, 137.29, 136.67, 136.39, 134.99, 134.75, 132.59, 132.31, 130.02, 129.74, 129.43, 129.19, 129.05, 126.63, 126.31, 51.89, 50.34, 46.93, 46.01, 34.18, 32.97. ESI-HRMS (*m*/*z*): [M + H]^+^ calcd for C_25_H_21_ClN_2_O_3_S_4_, 561.0123; found, 561.0116.

#### *N*-(4-Bromobenzyl)-*N*-(4-sulfamoylphenethyl)-4-(3-thioxo-3*H*-1,2-dithiol-4-yl)benzamide (**22**)

Compound **22** was obtained as an orange powder according to the general procedure reported earlier using **7** (1.1 equiv). Yield 56%; mp 65–66 °C. δ_H_ (400 MHz, DMSO-*d*_6_): 9.21 (s, 1H, =C*H*), 7.40 (m, 12H, Ar–*H*), 7.30 (s, 2H, exchange with D_2_O, SO_2_N*H*_2,_ overlap with signal at 7.40), 4.75 (s, 1.2H, C*H*_2_), 4.41 (s, 0.8H, C*H*_2_), 3.57 (m, 1H, C*H*_2_), 3.34 (m, 1H, C*H*_2_), 2.99 (m, 1H, C*H*_2_), 2.85 (m, 1H, C*H*_2_). δ_C_ (100 MHz, DMSO-*d*_6_): 214.13, 213.98, 171.14, 160.07, 147.77, 147.55, 143.69, 143.65, 142.80, 137.76, 137.59, 136.78, 136.67, 134.94, 134.75, 132.10, 131.95, 130.39, 129.72, 129.42, 126.62, 126.29, 121.07, 120.74, 51.97, 50.38, 49.38, 46.96, 46.01, 34.26, 34.03, 32.93. ESI-HRMS (*m*/*z*): [M + H]^+^ calcd for C_25_H_21_BrN_2_O_3_S_4_, 604.9618; found, 604.9621.

#### *N*-(4-Cyanobenzyl)-*N*-(4-sulfamoylphenethyl)-4-(3-thioxo-3*H*-1,2-dithiol-4-yl)benzamide (**23**)

Compound **23** was obtained as an orange powder according to the general procedure reported earlier using **8** (1.1 equiv). Yield 50%; mp 251–252 °C. δ_H_ (400 MHz, DMSO-*d*_6_): 9.22 (s, 1H, =C*H*), 7.50 (m, 12H, Ar–*H*), 7.30 (s, 2H, exchange with D_2_O, SO_2_N*H*_2,_ overlap with signal at 7.50), 4.87 (s, 1.3H, C*H*_2_), 4.54 (s, 0.7H, C*H*_2_), 3.60 (m, 1H, C*H*_2_), 3.40 (m, 1H, C*H*_2_), 3.01 (m, 1H, C*H*_2_), 2.87 (m, 1H, C*H*_2_). δ_C_ (100 MHz, DMSO-*d*_6_): 214.73, 214.51, 171.89, 171.77, 160.85, 160.67, 148.34, 148.13, 144.90, 143.39, 143.34, 137.05, 135.41, 133.75, 133.58, 130.33, 130.02, 129.38, 129.02, 128.89, 127.26, 126.88, 119.96, 119.76, 111.31, 110.95, 52.94, 51.47, 48.30, 46.90, 34.88, 33.54. ESI-HRMS (*m*/*z*): [M + H]^+^ calcd for C_26_H_21_N_3_O_3_S_4_, 552.0465; found, 552.0471.

#### *N*-(4-(Dimethylamino)benzyl)-*N*-(4-sulfamoylphenethyl)-4-(3-thioxo-3*H*-1,2-dithiol-4-yl)benzamide (**24**)

Compound **24** was obtained as an orange powder according to the general procedure reported earlier using **9** (1.1 equiv). Yield 57%; mp 185–186 °C. δ_H_ (400 MHz, DMSO-*d*_6_): 9.20 (s, 1H, =C*H*), 7.38 (m, 10H, Ar–*H*), 7.30 (s, 2H, exchange with D_2_O, SO_2_N*H*_2,_ overlap with signal at 7.38), 6.56 (m, 2H, Ar–*H*), 4.66 (s, 1H, C*H*_2_), 4.28 (s, 1H, C*H*_2_), 3.61 (s, 1H, C*H*_2_), 3.53 (s, 1H, C*H*_2_), 3.29 (m, 1H, C*H*_2_), 3.14 (m, 1H, C*H*_2_), 2.88 (m, 6H, 2 × C*H*_3_). δ_C_ (100 MHz, DMSO-*d*_6_): 214.12, 214.03, 170.89, 170.69, 160.21, 160.04, 150.32, 150.25, 147.80, 147.61, 143.86, 142.98, 142.70, 137.09, 134.74, 134.53, 129.82, 129.41, 128.51, 126.86, 126.47, 126.27, 125.27, 123.99, 113.00, 52.07, 49.48, 46.55, 45.31, 40.67, 34.10, 32.95. ESI-HRMS (*m*/*z*): [M + H]^+^ calcd for C_27_H_27_N_3_O_3_S_4_, 570.0935; found, 570.0929.

#### *N*-(4-Nitrobenzyl)-*N*-(4-sulfamoylphenethyl)-4-(3-thioxo-3*H*-1,2-dithiol-4-yl)benzamide (**25**)

Compound **25** was obtained as a yellow powder according to the general procedure reported earlier using **10** (1.1 equiv). Yield 45%; mp 223–224 °C. δ_H_ (400 MHz, DMSO-*d*_6_): 9.22 (s, 1H, =C*H*), 8.25 (d, *J* = 7.7 Hz, 2H, Ar–*H*), 7.56 (m, 7H, Ar–*H*), 7.30 (s, 2H, exchange with D_2_O, SO_2_N*H*_2,_ overlap with signal at 7.56), 7.12 (d, *J* = 7.3 Hz, 2H, Ar–*H*), 4.91 (s, 1.3H, C*H*_2_), 4.60 (s, 0.7H, C*H*_2_), 3.62 (m, 1H, C*H*_2_), 3.13 (m, 1H, C*H*_2_), 3.02 (m, 1H, C*H*_2_), 2.88 (m, 1H, C*H*_2_). δ_C_ (100 MHz, DMSO-*d*_6_): 214.15, 213.93, 171.29, 160.13, 147.76, 147.30, 147.15, 146.52, 143.26, 142.81, 142.44, 136.53, 136.43, 134.96, 134.87, 129.73, 129.42, 129.01, 128.66, 127.08, 126.65, 126.27, 124.18, 123.79, 52.16, 50.91, 47.52, 46.49, 34.30, 32.93. ESI-HRMS (*m*/*z*): [M + H]^+^ calcd for C_25_H_21_N_3_O_5_S_4_, 572.0364; found, 572.0369.

#### *N*-(2-Methoxy-4-nitrobenzyl)-*N*-(4-sulfamoylphenethyl)-4-(3-thioxo-3*H*-1,2-dithiol-4-yl)benzamide (**26**)

Compound **26** was obtained as an orange powder according to the general procedure reported earlier using **11** (1.1 equiv). Yield 44%; mp 190–191 °C. δ_H_ (400 MHz, DMSO-*d*_6_): 9.22 (s, 1H, =C*H*), 8.25 (d, *J* = 7.7 Hz, 2H, Ar–*H*), 7.56 (m, 11H, Ar–*H*), 7.29 (s, 2H, exchange with D_2_O, SO_2_N*H*_2,_ overlap with signal at 7.56), 4.78 (s, 1.2H, C*H*_2_), 4.46 (s, 0.8H, C*H*_2_), 4.04 (s, 1.7H, OC*H*_3_), 3.82 (s, 1.3H, OC*H*_3_), 3.61 (m, 1H, C*H*_2_), 3.46 (m, 1H, C*H*_2_), 2.99 (m, 1H, C*H*_2_), 2.88 (m, 1H, C*H*_2_). δ_C_ (100 MHz, DMSO-*d*_6_): 214.23, 214.04, 171.18, 160.24, 160.08, 157.66, 148.46, 148.09, 142.76, 136.78, 136.55, 134.86, 133.99, 133.18, 129.71, 129.36, 128.51, 126.67, 126.24, 116.15, 105.83, 56.70, 56.58, 51.11, 48.50, 46.59, 43.56, 34.49, 33.01. ESI-HRMS (*m*/*z*): [M + H]^+^ calcd for C_26_H_23_N_3_O_6_S_4_, 602.0469; found, 602.0473.

#### *N*-(4-Methoxybenzyl)-*N*-(4-sulfamoylphenethyl)-4-(3-thioxo-3*H*-1,2-dithiol-4-yl)benzamide (**27**)

Compound **27** was obtained as an orange powder according to the general procedure reported earlier using **12** (1.1 equiv). Yield 62%; mp 151–152 °C. δ_H_ (400 MHz, DMSO-*d*_6_): 9.20 (s, 1H, =C*H*), 7.33 (m, 12H, Ar–*H*), 7.31 (s, 2H, exchange with D_2_O, SO_2_N*H*_2,_ overlap with signal at 7.33), 4.71 (s, 1.1H, C*H*_2_), 4.34 (s, 0.9H, C*H*_2_), 3.74 (s, 3H, C*H*_3_), 3.53 (s, 1H, C*H*_2_), 3.34 (s, 1H, C*H*_2_), 2.95 (s, 1H, C*H*_2_), 2.83 (s, 1H, C*H*_2_). δ_C_ (100 MHz, DMSO-*d*_6_): 214.14, 213.99, 170.97, 170.81, 160.20, 160.07, 159.13, 158.99, 147.84, 147.61, 143.83, 142.92, 142.77, 136.94, 134.84, 134.61, 129.71, 129.42, 128.87, 126.76, 126.53, 126.27, 114.63, 114.51, 55.56, 51.98, 49.81, 46.64, 45.55, 34.13, 32.94. ESI-HRMS (*m*/*z*): [M + H]^+^ calcd for C_26_H_24_N_2_O_4_S_4_, 557.0618; found, 557.0611.

#### *N*-(3,4-Dimethoxybenzyl)-*N*-(4-sulfamoylphenethyl)-4-(3-thioxo-3*H*-1,2-dithiol-4-yl)benzamide (**28**)

Compound **28** was obtained as an orange powder according to the general procedure reported earlier using **13** (1.1 equiv). Yield 53%; mp 183–184 °C. δ_H_ (400 MHz, DMSO-*d*_6_): 9.20 (s, 1H, =C*H*), 7.69 (m, 4H, Ar–*H*), 7.33 (m, 3H, Ar–*H*), 7.30 (s, 2H, exchange with D_2_O, SO_2_N*H*_2,_ overlap with signal at 7.33), 7.03 (m, 3H, Ar–*H*), 6.71 (m, 1H, Ar–*H*), 4.71 (s, 1.1H, C*H*_2_), 4.35 (s, 0.9H, C*H*_2_), 3.58 (m, 1H, C*H*_2_), 3.35 (m, 1H, C*H*_2_), 3.32 (s, 3H, C*H*_3_), 3.30 (s, 3H, C*H*_3_), 2.97 (m, 1H, C*H*_2_), 2.83 (m, 1H, C*H*_2_). Δ_C_ (100 MHz, DMSO-*d*_6_): 214.14, 213.93, 170.99, 170.79, 160.24, 160.06, 149.33, 148.54, 147.79, 147.58, 146.10, 145.42, 143.84, 142.93, 142.73, 138.11, 136.94, 134.79, 134.59, 131.10, 130.45, 129.68, 129.59, 129.52, 129.40, 126.80, 126.50, 126.24, 112.32, 112.15, 55.96, 55.91, 52.22, 49.80, 46.91, 45.68, 39.95, 34.09, 32.98. ESI-HRMS (*m*/*z*): [M + H]^+^ calcd for C_27_H_26_N_2_O_5_S_4_, 587.0724; found, 587.0722.

#### *N*-(4-(Methylthio)benzyl)-*N*-(4-sulfamoylphenethyl)-4-(3-thioxo-3*H*-1,2-dithiol-4-yl)benzamide (**29**)

Compound **29** was obtained as an orange powder according to the general procedure reported earlier using **14** (1.1 equiv). Yield 57%; mp 161–162 °C. δ_H_ (400 MHz, DMSO-*d*_6_): 9.21 (s, 1H, =C*H*), 7.68 (m, 4H, Ar–*H*), 7.31 (m, 8H, Ar–*H*), 7.30 (s, 2H, exchange with D_2_O, SO_2_N*H*_2,_ overlap with signal at 7.31), 4.74 (s, 1.2H, C*H*_2_), 4.38 (s, 0.8H, C*H*_2_), 3.56 (m, 1H, C*H*_2_), 3.38 (m, 1H, C*H*_2_), 2.98 (m, 1H, C*H*_2_), 2.85 (m, 1H, C*H*_2_), 2.48 (s, 3H, C*H*_3_). δ_C_ (100 MHz, DMSO-*d*_6_): 214.13, 214.00, 171.03, 170.94, 160.26, 160.10, 147.78, 147.57, 143.75, 143.69, 142.86, 142.79, 137.85, 137.37, 136.82, 134.86, 134.66, 133.78, 129.71, 129.42, 128.93, 128.11, 126.70, 126.56, 126.27, 52.11, 50.09, 46.88, 45.76, 34.14, 32.96, 15.25, 15.16. ESI-HRMS (*m*/*z*): [M + H]^+^ calcd for C_26_H_24_N_2_O_3_S_5_, 573.0390; found, 573.0393.

#### *N*-(Naphthalen-1-ylmethyl)-*N*-(4-sulfamoylphenethyl)-4-(3-thioxo-3*H*-1,2-dithiol-4-yl)benzamide (**30**)

Compound **30** was obtained as an orange powder according to the general procedure reported earlier using **15** (1.1 equiv). Yield 64%; mp 116–117 °C. δ_H_ (400 MHz, DMSO-*d*_6_): 9.21 (s, 1H, =C*H*), 9.14 (s, 1H, =C*H*), 8.17 (d, *J* = 7.9 Hz, 1H, Ar–*H*), 7.96 (m, 2H, Ar–*H*), 7.53 (m, 11H, Ar–*H*), 7.30 (s, 2H, exchange with D_2_O, SO_2_N*H*_2,_ overlap with signal at 7.53), 7.07 (d, *J* = 7.8 Hz, 1H, Ar–*H*), 5.28 (s, 1.3H, C*H*_2_), 4.96 (s, 0.7H, C*H*_2_), 3.65 (m, 1H, C*H*_2_), 3.35 (m, 1H, C*H*_2_), 3.02 (m, 1H, C*H*_2_), 2.83 (m, 2H, C*H*_2_). δ_C_ (100 MHz, DMSO-*d*_6_): 214.15, 213.92, 171.08, 170.91, 160.20, 159.98, 147.83, 147.43, 143.83, 142.81, 136.83, 136.70, 135.08, 134.70, 133.97, 133.82, 133.01, 132.79, 131.96, 131.62, 130.87, 129.72, 129.43, 129.20, 128.51, 128.39, 126.97, 126.54, 126.41, 126.28, 126.06, 124.46, 123.84, 123.18, 50.68, 49.67, 46.87, 45.23, 34.00, 33.18. ESI-HRMS (*m*/*z*) [M + H]^+^ calcd for C_29_H_24_N_2_O_3_S_4_, 577.0669; found, 577.0665.

#### *N*-(Benzo[*b*]thiophen-3-ylmethyl)-*N*-(4-sulfamoylphenethyl)-4-(3-thioxo-3*H*-1,2-dithiol-4-yl)benzamide (**31**)

Compound **31** was obtained as an orange powder according to the general procedure reported earlier using **16** (1.1 equiv). Yield 55%; mp 113–114 °C. δ_H_ (400 MHz, DMSO-*d*_6_): 9.20 (s, 1H, =C*H*), 8.00 (m, 2H, Ar–*H*), 7.52 (m, 10H, Ar–*H*), 7.30 (s, 2H, exchange with D_2_O, SO_2_N*H*_2,_ overlap with signal at 7.52), 7.08 (d, *J* = 7.8 Hz, 1H, Ar–*H*), 5.07 (s, 1.3H, C*H*_2_), 4.69 (s, 0.7H, C*H*_2_), 3.67 (m, 1H, C*H*_2_), 3.34 (m, 1H, C*H*_2_), 3.00 (m, 1H, C*H*_2_), 2.84 (m, 1H, C*H*_2_). δ_C_ (100 MHz, DMSO-*d*_6_): 214.15, 213.98, 170.88, 170.27, 160.03, 142.80, 142.07, 140.47, 138.33, 136.78, 136.42, 134.68, 133.20, 132.41, 132.22, 129.67, 129.58, 129.50, 129.38, 126.78, 126.50, 126.26, 125.16, 124.87, 123.59, 123.43, 123.12, 122.30, 49.60, 47.92, 43.74, 41.60, 34.45, 33.10. ESI-HRMS (*m*/*z*): [M + H]^+^ calcd for C_27_H_22_N_2_O_3_S_5_, 583.0234; found, 583.0240.

#### *N*-Phenethyl-*N*-(4-sulfamoylphenethyl)-4-(3-thioxo-3*H*-1,2-dithiol-4-yl)benzamide (**32**)

Compound **32** was obtained as an orange powder according to the general procedure reported earlier using **17** (1.1 equiv). Yield 66%; mp 228–229 °C. δ_H_ (400 MHz, DMSO-*d*_6_): 9.20 (s, 1H, =C*H*), 7.42 (m, 13H, Ar–*H*), 7.31 (s, 2H, exchange with D_2_O, SO_2_N*H*_2,_ overlap with signal at 7.42), 3.73 (m, 2H, C*H*_2_), 3.48 (m, 2H, C*H*_2_), 2.86 (m, 4H, 2 x C*H*_2_). δ_C_ (100 MHz, DMSO-*d*_6_): 214.16, 170.73, 160.09, 147.88, 147.27, 146.74, 146.26, 143.92, 143.00, 142.73, 139.74, 138.74, 137.54, 137.32, 136.14, 134.47, 129.82, 129.71, 129.29, 129.16, 128.87, 127.34, 126.77, 126.43, 126.21, 52.35, 50.61, 46.36, 46.32, 34.54, 33.36, 26.43, 26.35. ESI-HRMS (*m*/*z*): [M + H]^+^ calcd for C_26_H_24_N_2_O_3_S_4_, 541.0669; found, 541.0672.

#### *N*-(2-Cyanoethyl)-*N*-(4-sulfamoylphenethyl)-4-(3-thioxo-3*H*-1,2-dithiol-4-yl)benzamide (**33**)

Compound **33** was obtained as an orange powder according to the general procedure reported earlier using **18** (1.1 equiv). Yield 41%; mp 154–155 °C. δ_H_ (400 MHz, DMSO-*d*_6_): 9.25 (s, 1H, =C*H*), 7.50 (m, 8H, Ar–*H*), 7.26 (s, 2H, exchange with D_2_O, SO_2_N*H*_2,_ overlap with signal at 7.50), 3.79 (m, 4H, 2 x C*H*_2_), 2.96 (m, 4H, 2 × C*H*_2_). δ_C_ (100 MHz, DMSO-*d*_6_): 214.12, 213.99, 171.19, 170.88, 160.36, 160.09, 147.78, 147.51, 142.98, 142.79, 142.18, 141.73, 136.66, 136.52, 134.95, 134.84, 133.00, 129.77, 129.41, 128.96, 128.38, 127.66, 126.96, 126.57, 126.25, 119.55, 119.39, 50.59, 49.71, 45.17, 42.27, 34.41, 33.35, 16.11, 16.02. ESI-HRMS (*m*/*z*): [M + H]^+^ calcd for C_21_H_19_N_3_O_3_S_4_, 490.0309; found, 490.0312.

### CA Inhibition

An Applied Photophysics stopped-flow instrument has been used for assaying the CA-catalyzed CO_2_ hydration activity.^38^ Phenol red (at a concentration of 0.2 mM) has been used as an indicator, working at the absorbance maximum of 557 nm, with 20 mM Hepes (pH 7.5) as a buffer and 20 mM Na_2_SO_4_ (for maintaining constant the ionic strength), following the initial rates of the CA-catalyzed CO_2_ hydration reaction for a period of 10–100 s. The CO_2_ concentrations ranged from 1.7 to 17 mM for the determination of the kinetic parameters and inhibition constants. For each inhibitor, at least six traces of the initial 5–10% of the reaction have been used for determining the initial velocity. The uncatalyzed rates were determined in the same manner and subtracted from the total observed rates. Stock solutions of inhibitor (0.1 mM) were prepared in distilled-deionized water and dilutions up to 0.01 nM were done thereafter with the assay buffer. Inhibitor and enzyme solutions were preincubated together for 15 min at r.t. before assay to allow for the formation of the E–I complex. The inhibition constants were obtained by nonlinear least-squares methods using PRISM 3 and the Cheng–Prusoff equation, as reported earlier,^39^ and represent the mean from at least three different determinations. The enzyme concentrations were in the range of 3–11 nM. All hCA isoforms were recombinant ones obtained in-house, as reported earlier.^32,33^

### Spectrophotometric Evaluation of H_2_S Release

Rat liver was homogenized in ice-cold 50 mM potassium phosphate buffer (pH 7.4).^41^ Optimal w/v ratios of 1/20 were determined from preliminary experiments (data not shown). The 3*H*-1,2-dithiole-3-thione derivatives were stocked as 30 mM solution in DMSO. The assay mixture (400 μL) contained the DTT compound incubated at 150 μM in phosphate buffer either in the presence or absence of a thiol derivative (such as l-cysteine and glutathione at 1.0 mM concentration) or in fresh rat liver homogenate (upon a further 1 to 10 dilution with phosphate buffer) in airtight vials at 37 °C for timed intervals (0–90 min). The released H_2_S concentration was measured as described previously.^45^ Briefly, after incubation, zinc acetate (1% w/v; 200 μL) was injected to trap the released H_2_S, followed by trichloroacetic acid (10% w/v, 200 μL) to precipitate the proteins present in the mixture and stop the H_2_S release reaction. Baseline H_2_S concentration was determined in incubates upon addition of trichloroacetic acid directly to the tissue homogenate in absence of the compound. Thus, *N*,*N*-dimethyl-*p*-phenylenediamine sulfate in HCl 5 M (20 mM; 200 μL) and FeCl_3_ in HCl 1.2 M (30 mM; 200 μL) were added to the mixture, and the absorbance of the formed methylene blue was measured after 30 min at 25 °C at 667 nm using a Varian Cary 300 UV–Visible spectrophotometer. The sample H_2_S concentration was calculated against a calibration curve of sodium sulfide in the concentration range 1–250 μM, and results, produced in triplicate, were plotted as released [H_2_S] versus time.

### In Vivo Antinociceptive Study

#### Animals

Sprague Dawley rats (Envigo, Varese, Italy) weighing 200–250 g at the beginning of the experimental procedure were used. Animals were housed in the Centro Stabulazione Animali da Laboratorio (CeSAL, University of Florence) and used at least 1 week after their arrival. Four rats were housed per cage (size 26 cm × 41 cm); animals were fed a standard laboratory diet and tap water ad libitum and kept at 23 ± 1 °C with a 12 h light/dark cycle (light at 7 a.m.). All animal manipulations were carried out according to the European Community guidelines for animal care [DL 116/92, application of the European Communities Council Directive of November 24, 1986 (86/609/EEC)]. The ethical policy of the University of Florence complies with the Guide for the Care and Use of Laboratory Animals of the U.S. National Institutes of Health (NIH Publication no. 85-23, revised 1996; University of Florence assurance number A5278-01). Formal approval to conduct the experiments described was obtained from the Italian Ministry of Health (no. 517/2017-PR) and from the Animal Subjects Review Board of the University of Florence. Experiments involving animals have been reported according to ARRIVE guidelines.^49^ All efforts were made to minimize animal suffering and to reduce the number of animals used.

#### Complete Freund’s Adjuvant-Induced Arthritis

Articular damage was induced by injection of Complete Freund’s Adjuvant (CFA; Sigma-Aldrich, St Louis, MO, USA), containing 1 mg/mL of heat-killed and dried *Mycobacterium tuberculosis* in paraffin oil and mannide monooleate, into the tibiotarsal joint.^50^ Briefly, the rats were lightly anesthetized with 2% isoflurane, the left leg skin was sterilized with 75% ethyl alcohol, and the lateral malleolus was located by palpation. A 28-gauge needle was then inserted vertically to penetrate the skin and turned distally for insertion into the articular cavity at the gap between the tibiofibular and tarsal bone until a distinct loss of resistance was felt. A volume of 50 μL of CFA was then injected (day 0). Control rats received 50 μL of saline solution (day 0) in the tibiotarsal joint.

#### Administration of Compounds

The hybrid derivatives **26**, **29**, **30,** and **31** (1, 10, and 30 mg/kg) were suspended in a 1% solution of carboxymethylcellulose (CMC) sodium salt and acutely *per os* administered starting from day 14 after CFA i.a. injection, when arthritis was well established. The H_2_S releaser **3** and CAI **15** were also administered alone or in coadministration at the equimolar dose of compound **30** 30 mg/kg (13.23 and 17.71 mg/kg, respectively). The antihypersensitivity effects were evaluated over time by the Paw pressure and Incapacitance tests.

#### Paw Pressure Test

The nociceptive threshold of rats was determined with an analgesimeter (Ugo Basile, Varese, Italy), according to the method described by Leighton et al.^51^ and Maresca et al.,^52^ (doi: 10.1111/jphp.12828). Briefly, a constantly increasing pressure was applied to a small area of the dorsal surface of the hind paw using a blunt conical probe by a mechanical device. Mechanical pressure was increased until vocalization or a withdrawal reflex occurred while rats were lightly restrained. Vocalization or withdrawal reflex thresholds were expressed in grams. Rats scoring below 40 g or over 75 g during the test before drug administration were rejected (25%).

#### Incapacitance Test

Weight-bearing changes were measured using an incapacitance apparatus (Linton Instrumentation, Norfolk, U.K.) to detect changes in postural equilibrium after a hind limb injury.^53,54^ Rats were trained to stand on their hind paws in a box with an inclined plane (65° from horizontal). This box was placed above the incapacitance apparatus. This allowed us to independently measure the weight that the animal applied on each hind limb. The value reported for each animal is the mean of five consecutive measurements. In the absence of hind limb injury, rats applied an equal weight on both hind limbs, indicating postural equilibrium, whereas an unequal distribution of weight on the hind limbs indicated a monolateral decreased pain threshold. Data are expressed as the difference between the weight applied to the limb contralateral to the injury and the weight applied to the ipsilateral limb (Δ weight).

#### Toxicity and the Irwin Test

Toxicity was evaluated after a single acute administration of **26**, **29**, **30,** and **31** at 30 mg/kg. Animals were observed for 24 h. For the Irwin test, each rat was individually placed in a transparent cage (26 × 41 cm), and 26 neurobehavioral or physiological parameters were systematically assessed according to Irwin (1968)^55^ and already reported by Berrino and colleagues in similar studies.^30^ Behavioral, autonomic, and neurological manifestations produced by compound administration in rats were evaluated: motor displacement, motor reflexes, stereotypies, grooming, reaction to painful or environmental stimuli (analgesia and irritability), startle response, secretions, excretions, respiratory movements, skin color and temperature, piloerection, exophthalmos, eyelid and corneal reflexes, muscle tone, ataxia, tremors, head twitches, jumps, convulsions, Straub tail, and other signs or symptoms. For postural reflexes (righting reflex) and other signs such as piloerection, exophthalmia (exaggerated protrusion of the eyeball), ataxia, tremors, and Straub tail, only presence or absence was recorded. Skin color was evaluated qualitatively (pale, red, or purple); other signs were evaluated semiquantitatively, according to the observer’s personal scale (0 to +4, −4 to 0, or −4 to +4). The terms sedation and excitation express the final interpretation of a group of signs: reduced motor activity, reduced startle response, eyelid ptosis, and reduced response to manual manipulation, for the former; and increased motor activity, increased startle response, increased response to manual manipulation, and exophthalmia, for the latter. Hyperactivity includes running, jumping, and attempting to escape from the container. Trained observers were not informed about the specific treatment of each animal group carried out in this test.

#### Liver Collection

At the end of the behavioral measurements, control animals were sacrificed by decapitation. The liver was collected and immediately frozen in liquid nitrogen to determine the release of H_2_S by spectrophotometric analysis as described above.

#### Statistical Analysis

Behavioral measurements were performed on eight rats for each treatment carried out in two different experimental sets. Results were expressed as the mean (SEM) with a one-way analysis of variance. A Bonferroni’s significant difference procedure was used as a *post hoc* comparison. *P*-values of <0.05 or <0.01 were considered significant. Data were analyzed using the Origin 9 software (OriginLab, Northampton, MA, USA).

## Supporting Information Available

SMILES representation of compounds (CSV)
CA inhibition profile of intermediates **4–18**, H_2_S release kinetic parameters, additional in vivo data, and HPLC chromatograms (PDF)

## Abbreviations

DCM: dichloromethane
DMAP: 4-dimethylaminopyridine
DMF: *N*,*N*-dimethylformamide
DMSO: dimethylsulfoxide
EDC·HCl: *N*-(3-dimethylaminopropyl)-*N*′-ethylcarbodiimide hydrochloride
Et_2_O: diethyl ether
EtOAc: ethyl acetate
EtOH: ethanol
MeOH: methanol
NaBH_4_: sodium borohydride
NaOH: sodium hydroxide
o.n.: overnight
r.t.: room temperature
Et_3_N: triethylamine
TFA: trifluoroacetic acid
