# Supplementary material for: Development of Hydrogen Sulfide-Releasing Carbonic Anhydrases IX- and XII-Selective Inhibitors with Enhanced Antihyperalgesic Action in a Rat Model of Arthritis
Source: J Med Chem. 2022 Sep 19;65(19):13143–57. doi: 10.1021/acs.jmedchem.2c00982 (PMC9574929; doi:10.1021/acs.jmedchem.2c00982)
Supplement: Supplementary file 2 — jm2c00982_si_002.pdf [file jm2c00982_si_002.pdf]

SUPPORTING INFORMATION

Development of hydrogen sulfide-releasing carbonic anhydrases IX and XII selective inhibitors with enhanced antihyperalgesic action in a rat model of arthritis

Alessandro Bonardi,[a†] Laura Micheli,[b†] Lorenzo Di Cesare Mannelli,[b] Carla Ghelardini,[b] Paola Gratteri,[a] Alessio Nocentini,[a*] Claudiu T. Supuran [a*]

[a] *Department of NEUROFARBA – Pharmaceutical and Nutraceutical section, University of Firenze, via Ugo Schiff 6, 50019 Sesto Fiorentino (Florence), Italy.*

[b] *Department NEUROFARBA – Section of Pharmacology and Toxicology, University of Florence, viale Gaetano Pieraccini 6, 50139 Firenze (Florence), Italy.*

† *These authors contribute equally*

email: alessio.nocentini@unifi.it (AN); claudiu.supuran@unifi.it (CTS).

CA inhibition data of sulfonamides 4-18 S2

H_2S release kinetic details S3

Irwin test S4

HPLC chromatograms S5

Table S1. Inhibition data of human CA isoforms hCA I, II, IV, IX, and XII with sulfonamides **4-18** reported here and the standard sulfonamide inhibitor acetazolamide (AAZ) by a stopped-flow CO2 hydrase assay.[31]

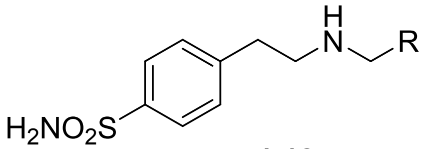

4-18

Cmpd	R	K_I (nM)[a]				
		**hCA I**	**hCA II**	**hCA IV**	**hCA IX**	**hCA XII**
**4**[b]	C_6H_5	95.3	98.4	2854.4	78.1	65.4
**5**[b]	4-F-C_6H_4	112.8	78.5	1196.7	56.5	60.1
**6**	4-Cl-C_6H_4	136.3	115.1	3678.1	67.4	75.2
**7**	4-Br-C_6H_4	213.8	189.5	5239.3	86.4	83.6
**8**	4-CN-C_6H_4	91.4	70.2	1784.4	75.4	52.9
**9**	4-N$(CH_3)_2$-C_6H_4	82.3	87.4	961.2	96.7	46.2
**10**[b]	4-NO_2-C_6H_4	224.3	120.9	1685.3	89.2	77.4
**11**	2-OCH_3,4-NO_2-C_6H_3	145.1	47.3	1862.1	59.7	95.2
**12**	4-OCH_3-C_6H_4	97.2	61.3	1445.7	90.4	57.2
**13**	3,4-diOCH_3-C_6H_3	88.6	75.2	1103.8	103.4	51.0
**14**	4-SCH_3-C_6H_4	122.2	94.7	2131.2	111.9	88.6
**15**	naphth-1-yl	261.2	177.5	3012.6	45.3	54.7
**16**	benzo[b]thiophen-3-yl	373.8	201.6	7241.3	70.5	69.8
**17**[b]	$CH_2C_6H_5$	278.4	89.1	3587.4	101.9	104.3
**18**[b]	CH_2CN	105.3	153.7	5547.2	104.7	113.2
**AAZ**	-	250	12	74	25	5.7

a. Mean from three different assays, by a stopped-flow technique (errors were in the range of ±5−10% of the reported values).
b. Data reported in Bonardi et al. [29,30]

Table S2. Selectivity index (*SI*) of sulfonamides **4-18** and standard acetazolamide (AAZ) against the antiinflammatory human CA isoforms IV, IX, and XII versus the ubiquitous cytosolic ones CA I and II.

	SI						
**Cmpd**	**CA I/II**	**CA I/IV**	**CA II/IV**	**CA I/IX**	**CA II/ IX**	**CA I/XII**	**CA II/XII**
**4**	0.9	0.03	0.03	1.2	1.3	1.5	1.5
**5**	1.4	0.09	0.06	2.0	1.4	1.9	1.3
**6**	1.2	0.04	0.03	2.0	1.7	1.8	1.5
**7**	1.1	0.04	0.04	2.5	2.2	2.6	2.3
**8**	1.3	0.05	0.04	1.2	0.9	1.7	1.3
**9**	0.9	0.09	0.09	0.9	0.9	1.8	1.9
**10**	1.9	0.1	0.07	2.5	1.4	2.9	1.6
**11**	3.1	0.08	0.03	2.4	0.8	1.5	0.5
**12**	1.6	0.07	0.04	1.1	0.7	1.7	1.1
**13**	1.2	0.08	0.07	0.9	0.7	0.9	1.5
**14**	1.3	0.05	0.04	1.1	0.8	1.4	1.1
**15**	1.5	0.08	0.06	5.8	4.0	4.8	3.2
**16**	1.9	0.05	0.03	5.3	2.8	5.3	2.8
**17**	3.1	0.08	0.02	2.7	0.08	2.6	0.9
**18**	0.7	0.01	0.02	1.0	1.5	0.9	1.4
**AAZ**	21.0	3.4	0.17	10.0	0.5	43.9	2.2

Table S3. H_2S-releasing Cmax and half-lives of compounds **3**, **26**, **29**, **30**, **31**.

**cmpd**	**C_{max}**	**t/2 (min)**
**3**	12.1	3.1
**26**	7.9	4.9
**29**	8.2	4.6
**30**	8.0	5.0
**31**	8.4	4.3

Table S4. Irwin test.

Hybrid	**26**	**29**	**30**	**31**	**Limits**
Dose	30 mg/kg	30 mg/kg	30 mg/kg	30 mg/kg	
Behaviour					
Spontaneous activity	4	4	4	4	4 - 0
Passivity	0	0	0	0	0 - 4
Cleaning	0	0	0	0	4 - 0
Curiosity	4	4	4	4	4 - 0
Reactivity	4	4	4	4	4 - 0
Vocalization	0	0	0	0	0 - 4
S.N.C. excitement					
Straub tail	0	0	0	0	0 - 4
Tremors	0	0	0	0	0 - 4
Convulsions	0	0	0	0	4 - 0
Movement					
Ataxia	0	0	0	0	0 - 4
Stereotipies	0	0	0	0	0 - 4
Straightening reflex	4	4	4	4	4 - 0
Muscolar tone					
Physical strenght	4	4	4	4	4 – 0
Reflexes					
Palpebral reflex	4	4	4	4	4 - 0
Autonomic signes					
Piloerection	0	0	0	0	0 - 4
Exolphthalmos	0	0	0	0	0 - 4
Cyanosis	0	0	0	0	0 - 4
Flush	0	0	0	0	0 - 4
Pallor	0	0	0	0	0 - 4
Palpebral opening	4	4	4	4	4 - 0
Salivation	0	0	0	0	0 - 4
Lacrimation	0	0	0	0	0 - 4
Hypo-hyperthermia	0	0	0	0	-4/+4
Writhing	0	0	0	0	0 - 4
Toxicity					
Immediate death	0	0	0	0	0 - 4
Delayed death (48 h)	0	0	0	0	0 - 4

The chromatographic profiles of LC-DAD analysis and a representative UV spectrum of compounds **19-33** are reported in Figures S1-S15.

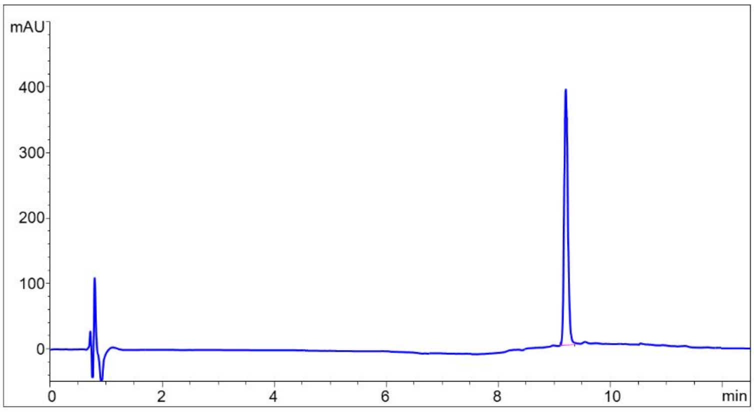

Figure S1. Chromatographic profile of **19** monitored at λ=230 nm.

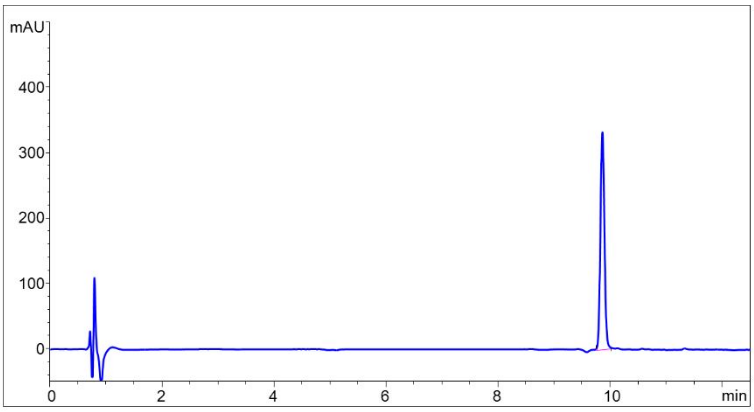

Figure S2. Chromatographic profile of **20** monitored at λ=230 nm.

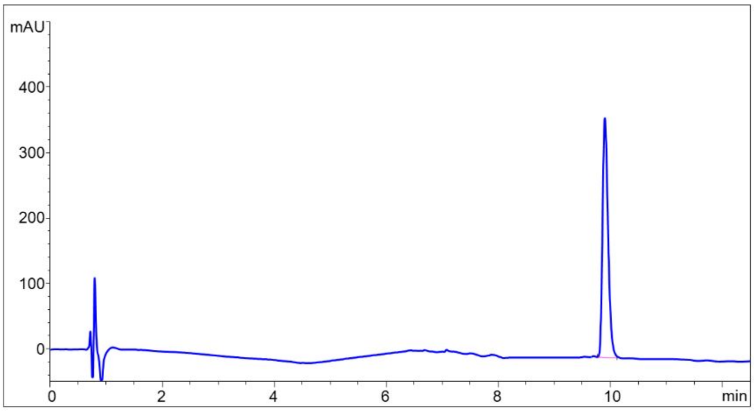

Figure S3. Chromatographic profile of **21** monitored at λ=230 nm.

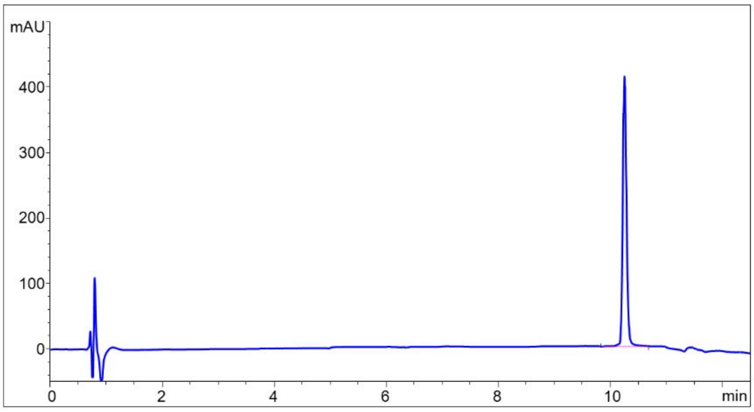

Figure S4. Chromatographic profile of **22** monitored at λ=230 nm.

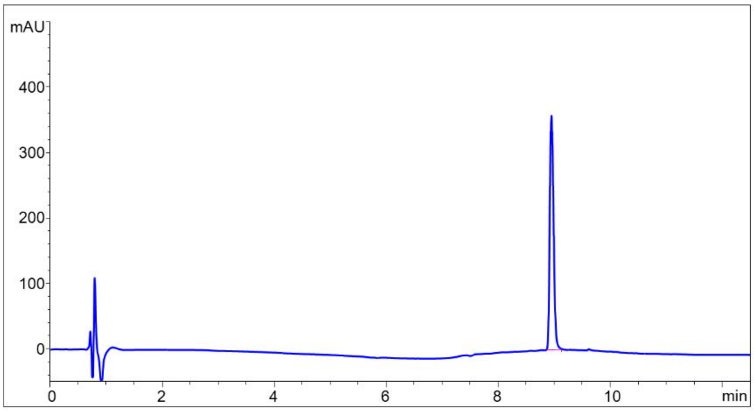

Figure S5. Chromatographic profile of **23** monitored at λ=230 nm.

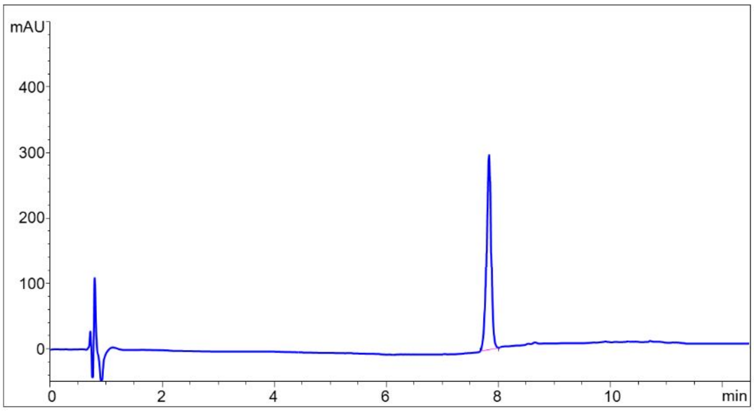

Figure S6. Chromatographic profile of **24** monitored at λ=230 nm.

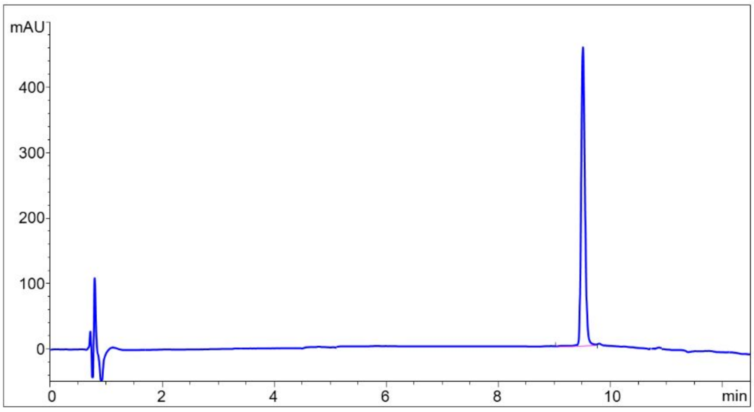

Figure S7. Chromatographic profile of **25** monitored at λ=230 nm.

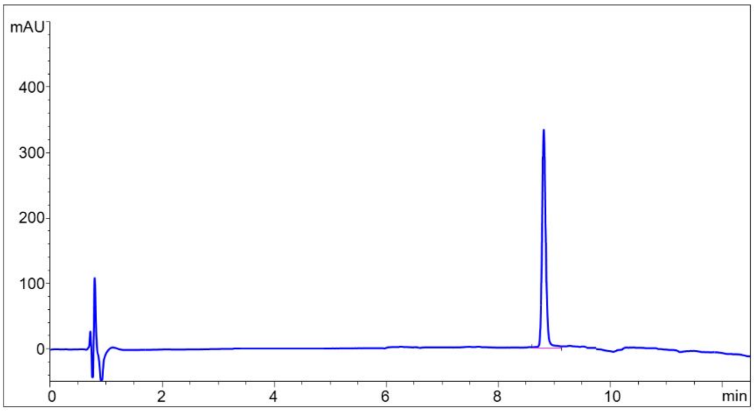

Figure S8. Chromatographic profile of **26** monitored at λ=230 nm.

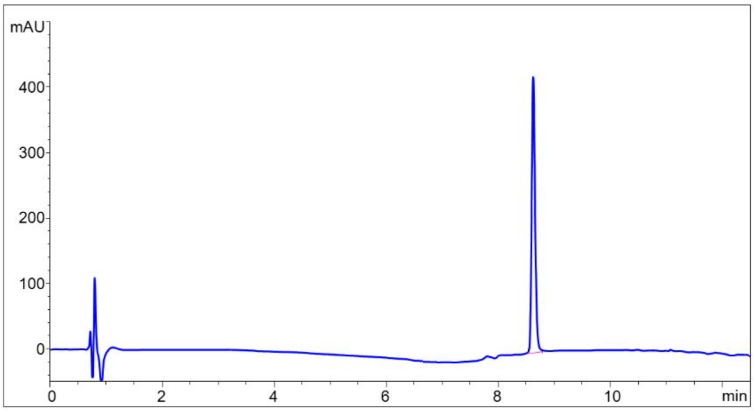

Figure S9. Chromatographic profile of **27** monitored at λ=230 nm.

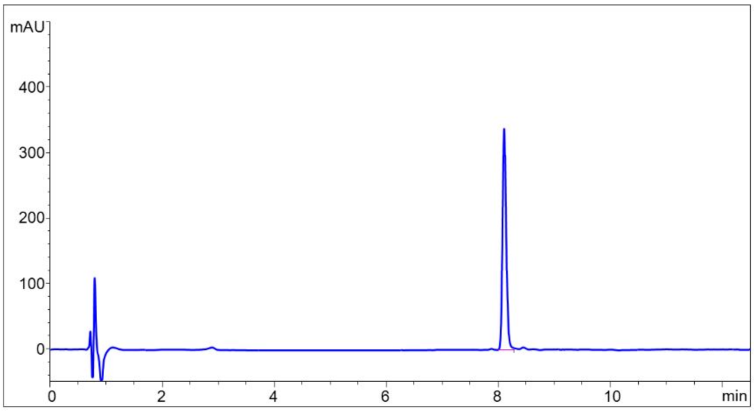

Figure S10. Chromatographic profile of **28** monitored at λ=230 nm.

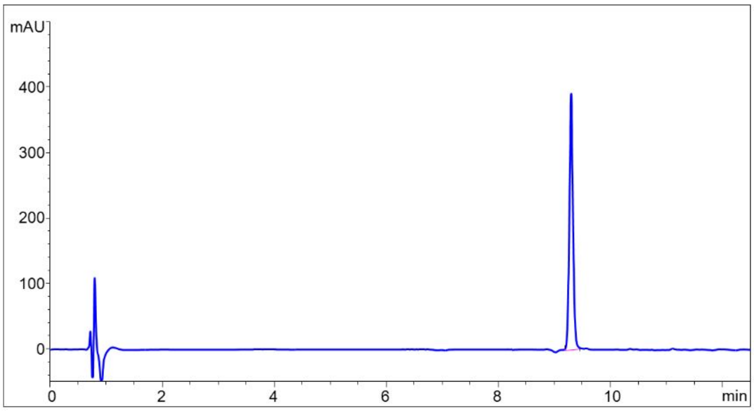

Figure S11. Chromatographic profile of **29** monitored at λ=230 nm.

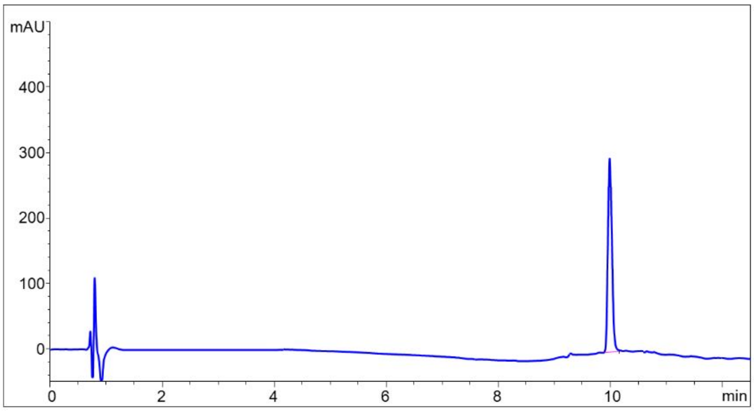

Figure S12. Chromatographic profile of **30** monitored at λ=230 nm.

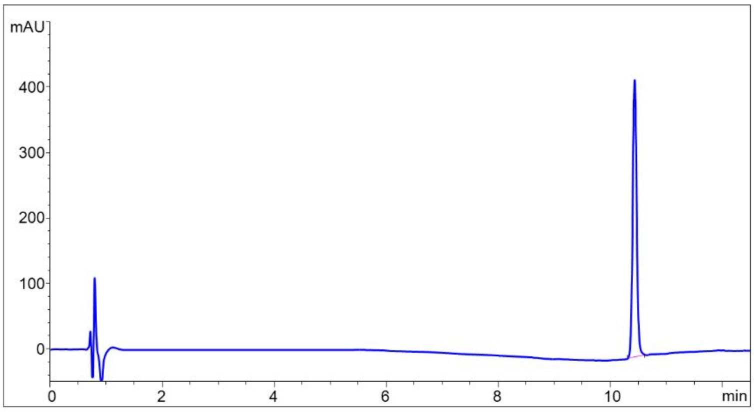

Figure S13. Chromatographic profile of **31** monitored at λ=230 nm.

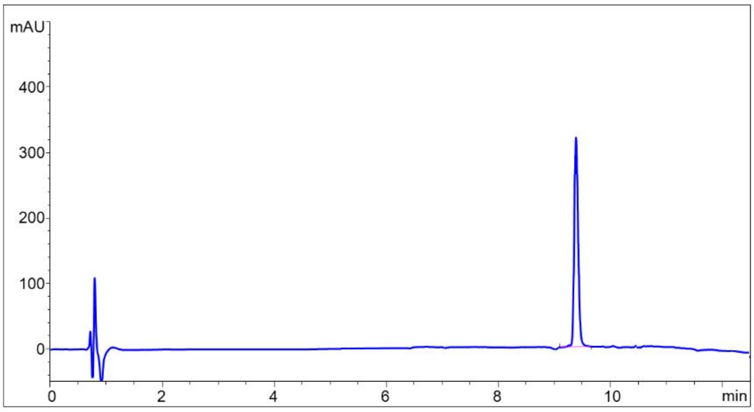

Figure S14. Chromatographic profile of **32** monitored at λ=230 nm.

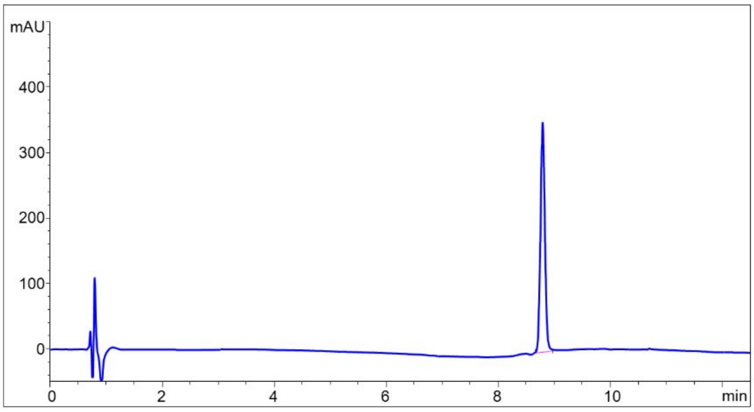

Figure S15. Chromatographic profile of **33** monitored at λ=230 nm.
